# Crystal structures and hydrogen-bonding analysis of a series of benzamide complexes of zinc(II) chloride

**DOI:** 10.1107/S2056989021008264

**Published:** 2021-08-17

**Authors:** Elizabeth Tinapple, Sam Farrar, Dean H. Johnston

**Affiliations:** a Department of Chemistry, Otterbein University, Westerville, OH 43081, USA

**Keywords:** ionic co-crystal, co-crystal salt, zinc(II) chloride, benzamide, tolu­amide

## Abstract

Ionic co-crystals are co-crystals between organic mol­ecules and inorganic salt coformers. Co-crystals of pharmaceuticals are of inter­est to help control polymorph formation and potentially improve stability and other physical properties. We describe the preparation, crystal structures, and hydrogen bonding of five different 2:1 benzamide or tolu­amide/zinc(II) chloride co-crystal salts, namely, bis­(benzamide-κ*O*)di­chlorido­zinc(II), [ZnCl_2_(C_7_H_7_NO)_2_], di­chlor­ido­bis­(2-methyl­benzamide-κ*O*)zinc(II), [ZnCl_2_(C_8_H_9_NO)_2_], di­chlorido­bis­(3-methyl­benzamide-κ*O*)zinc(II), [ZnCl_2_(C_8_H_9_NO)_2_], di­chlorido­bis­(4-methyl­benzamide-κ*O*)zinc(II), [ZnCl_2_(C_8_H_9_NO)_2_], and di­chlorido­bis­(4-hy­droxy­benzamide-κ*O*)zinc(II), [ZnCl_2_(C_7_H_7_NO_2_)_2_]. All of the complexes contain hydrogen bonds between the amide N—H group and the amide carbonyl oxygen atoms or the chlorine atoms, forming extended networks.

## Abstract 2

Five new bis­(aryl­amide)­dichlorido­zinc(II) complexes have been prepared and characterized. All of the complexes contain hydrogen bonds between the amide N—H group and the amide carbonyl oxygen atoms or the chlorine atoms, forming extended networks.

## Chemical context   

Ionic co-crystals, formed from the combination of inorganic salts and organic mol­ecules, are of inter­est for their ability to promote or stabilize crystal forms of organic or pharmaceutical mol­ecules (Braga *et al.*, 2011 ▸, 2018 ▸). The chloride salts of magnesium, calcium, and strontium have been shown to form an extensive range of structure types when co-crystallized with drug mol­ecules such as piracetam (Braga *et al.*, 2011 ▸; Song *et al.*, 2018 ▸), etiracetam and levitiracetam (Song *et al.*, 2019 ▸, 2020 ▸), and nicotinamide and isonicotinamide (Braga *et al.*, 2011 ▸; Song *et al.*, 2020 ▸). Sodium bromide and sodium iodide form ionic co-crystals with carbamazepine (Buist & Kennedy, 2014 ▸). More recently, it has been shown that co-crystallization with ionic salts can produce chirally resolved forms when combining lithium halides with l- and dl-histidine (Braga *et al.*, 2016 ▸), magnesium chloride with *RS*-oxiracetam (Shemchuk *et al.*, 2020 ▸), and zinc chloride with *RS*-etiracetam (Shemchuk *et al.*, 2018 ▸). Co-crystallization of nefiracetam with zinc chloride produced products with improved solubility and dissolution rates (Buol *et al.*, 2020 ▸).
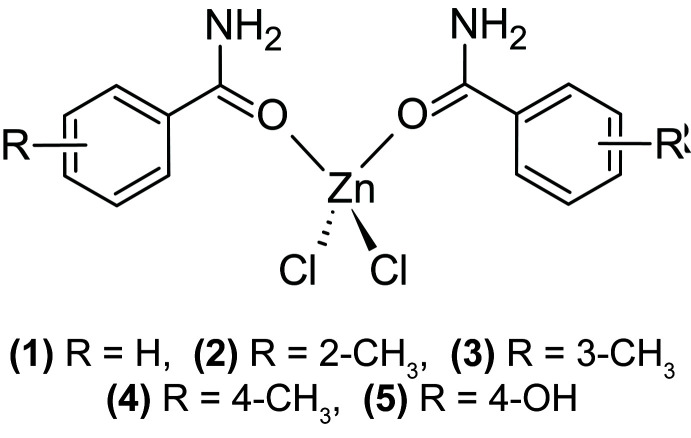


The current study was undertaken to explore the preparation of ionic co-crystals (alternatively termed co-crystal salts; Grothe, *et al.*, 2016 ▸) using zinc chloride combined with various organic amides (specifically benzamide, 4-hy­droxy­benzamide, and tolu­amide) that can serve as models of pharmaceutical mol­ecules.

## Structural commentary   

Five new zinc complexes, (**1**) through (**5**), have been prepared and structurally characterized. All five complexes are 2:1 O-bonded aryl amide:ZnCl_2_ complexes with approximately tetra­hedral zinc(II) centers. The complexes crystallize in five different space groups and form hydrogen-bonding inter­actions between the amide N—H groups and either an amide oxygen or a zinc-bound chlorido ligand.

**Figure 1:**
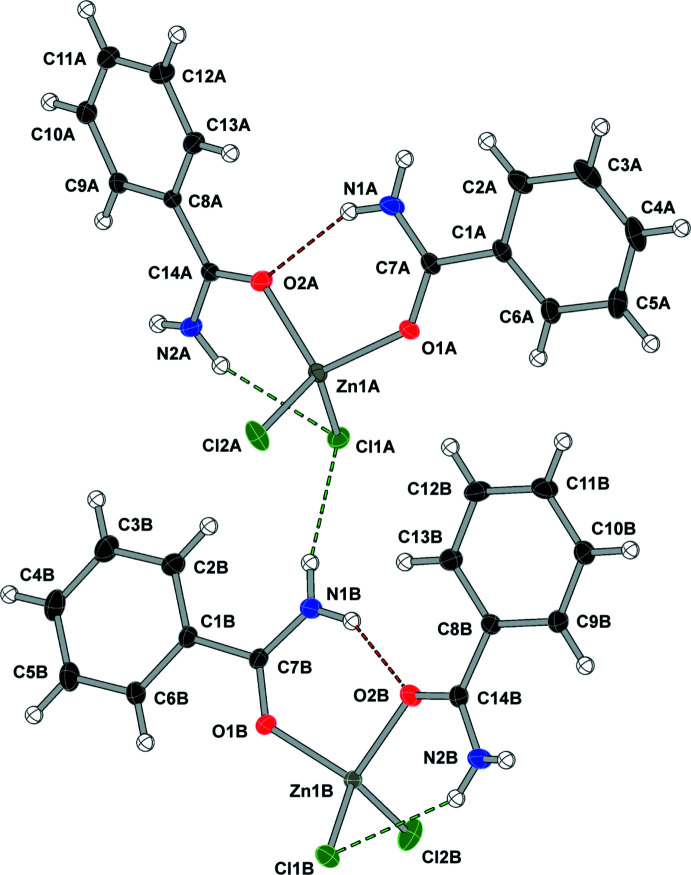
Displacement ellipsoid (50%) diagram and atom-numbering scheme of the two independent mol­ecules in (**1**). N—H⋯O contacts are shown in red and N—H⋯Cl contacts are shown in green.

Compound (**1**), bis­(benzamide-κ*O*)di­chlorido­zinc(II), [ZnCl_2_(C_7_H_7_NO)_2_], crystallizes in the *P*2_1_/*n* space group with two independent mol­ecules in the asymmetric unit and displays one N—H⋯O and one N—H⋯Cl intra­molecular hydrogen bond in each mol­ecule (see Fig. 1 ▸ and Table 1 ▸). A search for non-crystallographic symmetry using *PLATON* (Spek, 2020 ▸) shows the two independent zinc complexes are related by a rotation of −173.2° and translation by 7.232 Å along the vector [1.000 0.101 0.992]. Alignment of the two residues gave a weighted r.m.s. fit of 0.330 Å.

**Figure 2:**
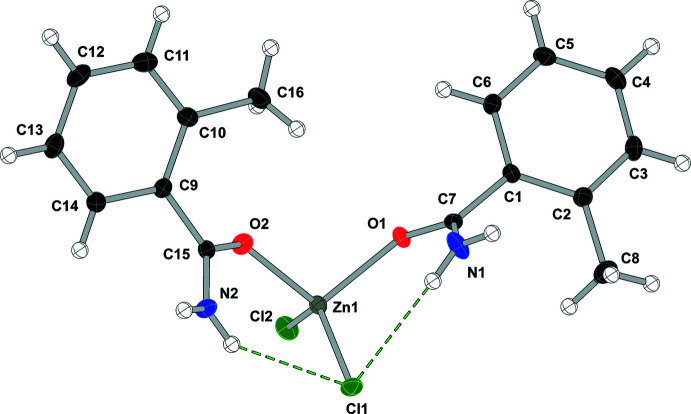
Displacement ellipsoid (50%) diagram and atom-numbering scheme for (**2**). N—H⋯Cl contacts are shown in green.

**Figure 3:**
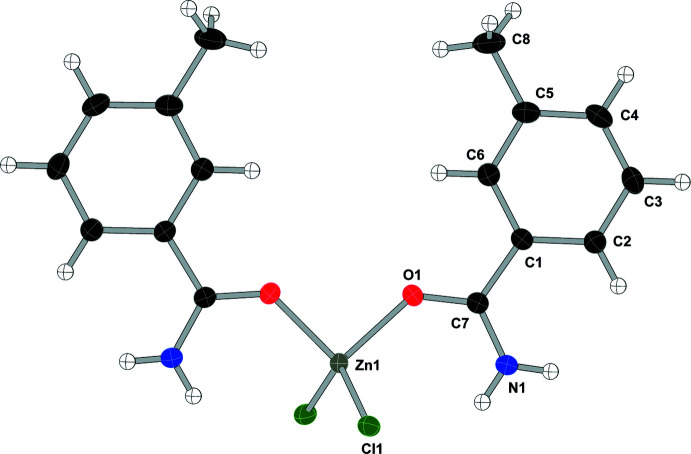
Displacement ellipsoid (50%) diagram and atom-numbering scheme for (**3**). The minor component of the disordered methyl group is not shown for clarity.

**Figure 4:**
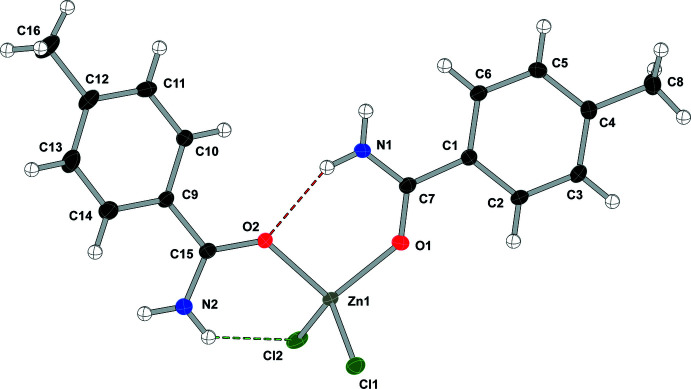
Displacement ellipsoid (50%) diagram and atom-numbering scheme for (**4**). The N—H⋯O contact is shown in red and the N—H⋯Cl contact is shown in green.

As shown in Fig. 2 ▸, compound (**2**), di­chlorido­bis­(2-methyl­benzamide-κ*O*)zinc(II), [ZnCl_2_(C_8_H_9_NO)_2_], displays two intra­molecular N—H⋯Cl hydrogen bonds to one chlorine atom (see Table 2 ▸) and crystallizes in the *P*2_1_ space group. Compound (**3**), di­chlorido­bis­(3-methyl­benzamide-κ*O*)zinc(II), [ZnCl_2_(C_8_H_9_NO)_2_], crystallizes in the *C*2/*c* space group with the zinc atom lying on the twofold axis (see Fig. 3 ▸) and, unlike the other compounds in this study, compound (**3**) does not form any intra­molecular hydrogen bonds. Compound (**4**), di­chlorido­bis­(4-methyl­benzamide-κ*O*)zinc(II), [ZnCl_2_(C_8_H_9_NO)_2_], crystallizes in the *P*2_1_/*c* space group and compound (**5**), di­chlorido­bis­(4-hy­droxy­benzamide-κ*O*)zinc(II), [ZnCl_2_(C_7_H_7_NO_2_)_2_], crystallizes in the *Cc* space group and both compounds form two intra­molecular hydrogen bonds, one N—H⋯O and one N—H⋯Cl, similar to the inter­actions found in compound (**1**) (see Figs. 4 ▸ and 5 ▸ and Tables 4 ▸ and 5 ▸).

A comparison of selected bond lengths and bond angles for all five complexes is given in Table 6 ▸. The average zinc–chlorine distance of 2.224 (13) Å compares well with the average of 2.22 (2) Å observed for 27 similar four-coordinate ZnCl_2_ *L*_2_ complexes (with *L* = carbonyl oxygen donating ligand) found in a search of the CSD (Version 5.42, May 2021; Groom *et al.*, 2016 ▸). A similar agreement is found for the zinc–oxygen distance with both averages at 1.98 (2) Å. The bond angles in the complexes in this study display an average Cl—Zn—Cl angle of 117 (5)° and an average O—Zn—O angle of 101 (3)°, again quite close to the average angles of 119 (4) and 100 (7)° for the set of comparable mol­ecules.

## Supra­molecular features   

**Figure 6:**
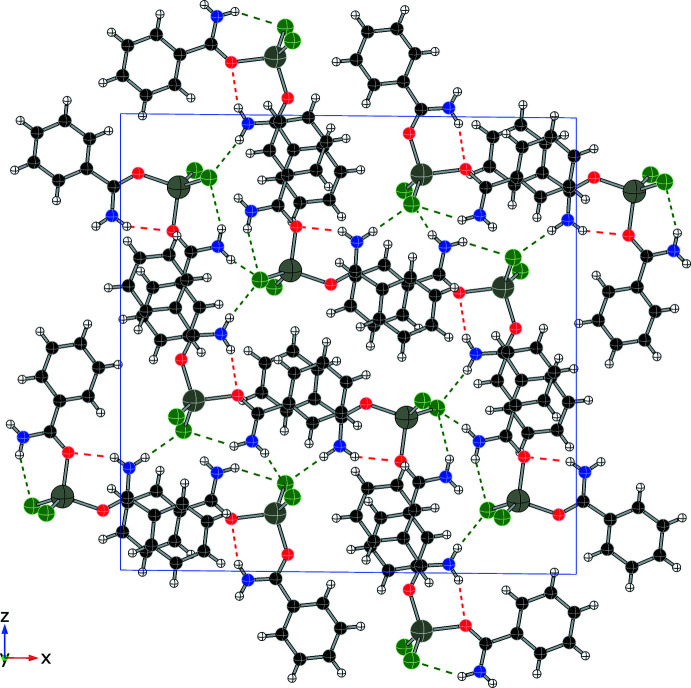
Packing diagram of (**1**) (viewed along *b*) showing N—H⋯O contacts (red) and N—H⋯Cl contacts (green).

**Figure 7:**
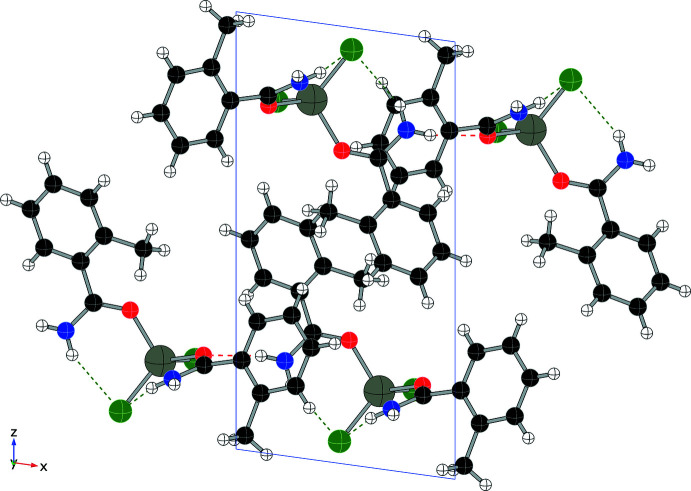
Packing diagram of (**2**) (viewed along *b*) showing N—H⋯O contacts (red) and N—H⋯Cl contacts (green).

**Figure 8:**
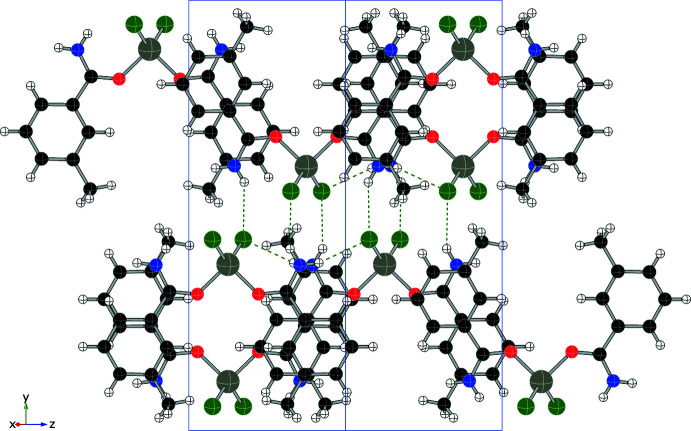
Packing diagram of (**3**) (viewed along [101]) showing N—H⋯Cl contacts (green). The minor component of the disordered methyl group is not shown for clarity.

**Figure 9:**
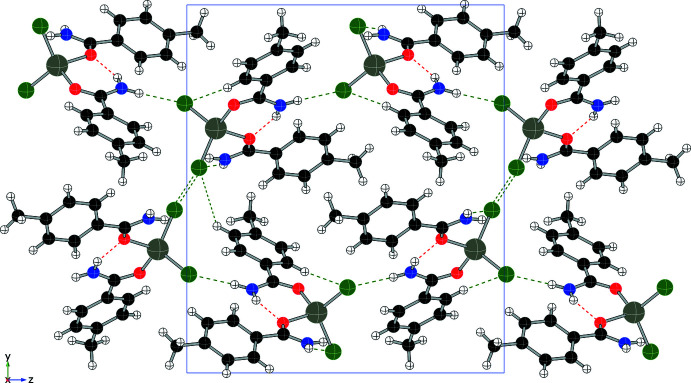
Packing diagram of (**4**) (viewed along *a*) showing N—H⋯O contacts (red) and N—H⋯Cl contacts (green).

**Figure 10:**
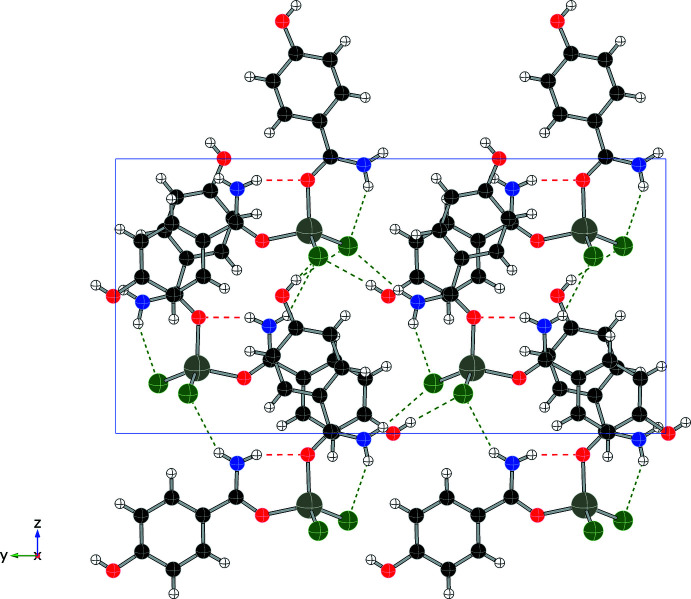
Packing diagram of (**5**) (viewed along *a*) showing N—H⋯O contacts (red) and N—H⋯Cl contacts (green).

Each compound displays a unique hydrogen-bonding network, consisting primarily of N—H⋯O and N—H⋯Cl inter­actions, summarized in Table 1 ▸ through 5. In addition to four intra­molecular hydrogen bonds, compound (**1**) forms four N—H⋯Cl inter­molecular hydrogen bonds (two from each independent mol­ecule), forming an extended network as shown in Fig. 6 ▸ and summarized in Table 1 ▸. Compound (**2**) also utilizes N—H bonds in hydrogen-bonding inter­actions, two intra­molecular and two inter­molecular, to form layers within the structure (see Fig. 7 ▸ and Table 2 ▸). Only inter­molecular N—H⋯Cl hydrogen bonds are found in compound (**3**) (shown in Fig. 8 ▸, two inter­actions per asymmetric unit, four per mol­ecule, see Table 3 ▸) and they combine to form chains that run parallel to the *c* axis. Compound (**4**) forms two N—H⋯Cl inter­molecular contacts in addition to the two intra­molecular hydrogen bonds, resulting in a complex set of layers that run perpendicular to the *b* axis (see Fig. 9 ▸ and Table 4 ▸). The addition of the 4-hy­droxy group in compound (**5**) results in the greatest number of hydrogen bonds among this set of complexes, as shown in Fig. 10 ▸ and summarized in Table 5 ▸, with two N—H⋯Cl and three O—H⋯Cl inter­molecular inter­actions per mol­ecule.

Compounds (**1**), (**3**), and (**5**) form π–π inter­actions between the benzene rings of the benzamide or tolu­amide groups as summarized in Table 7 ▸. No significant π–π inter­actions were found for compounds (**2**) or (**4**).

## Database survey   

A search of the CSD (Version 5.42, May 2021; Groom *et al.*, 2016 ▸) produced a relatively small number of amide-coordinated zinc(II)chloride complexes. One of the earliest is a di­chlorido­bis­(dma)zinc(II) complex (CSD refcode: DMAMZN10; Herceg & Fischer, 1974 ▸; dma = *N*,*N*-di­methyl­acetamide). The similar di­chlorido­bis­(dmf)zinc(II) (KOBWIH; Suzuki *et al.*, 1991 ▸; dmf = *N*,*N*-di­methyl­formamide) has also been reported. Edwards *et al.* (1999 ▸, 1998 ▸) investigated the structures of a series of Zn*X*_2_ *L*_2_ complexes that included *L* = dmf and *X* = Br and I (FIQBEM, FEXWIO, respectively), the latter of which undergoes a reversible phase transition at 228 K (Edwards *et al.*, 1998 ▸). A similar study (Turnbull *et al.*, 2000 ▸) compared the structures of Zn*X*_2_(dma)_2_ where *X* = Cl, Br, I (DMAMZN11, CAHWEO, CAHWAK, respectively). As part of a larger study, Smirnov *et al.* (2014 ▸) prepared and crystallographically characterized di­methyl­urea complexes of zinc(II)chloride and zinc(II)bromide (ZZZSAG01, COQXIR) along with bis­(piperidine-1-carboxamide) zinc(II)halide complexes (COQWOW, COQVIP), all of which display intra­molecular N—H⋯O hydrogen bonding similar to that observed in this study.

A number of zinc(II) iodide complexes, ZnI_2_ *L*_2_, have been prepared with simple amide ligands, including urea (ACAQAW; Furmanova *et al.*, 2001 ▸), acetamide (VIDBOA; Savinkina *et al.*, 2007 ▸), and formamide (DIYGUO; Savinkina *et al.*, 2008 ▸). Savinkina *et al.* (2009 ▸) have also prepared a series of ZnI_2_ *L*_2_ complexes with *L* = di­methyl­urea (VUCTUJ), thio­acetamide (VUCTOD), and benzamide (VUCVAR).

Three structural studies have prepared zinc(II)chloride complexes with pharmaceutically relevant mol­ecules. Sultana *et al.* (2016 ▸) prepared bis­(4′-meth­oxy­acetanilide)di­chlorido­zinc(II) (EQIGOC). Di­chlorido­bis­(nicotinamide)­zinc(II) has also been studied (WUKZAD; İde *et al.*, 2002 ▸) but differs from the structures in this report in that the two nicotinamide ligands are N-bonded through the ring nitro­gen instead of the amide oxygen. Buol *et al.* (2020 ▸) describe the preparation and crystal structures of co-crystals obtained from the co-crystallization of nefiracetam with zinc(II)chloride, producing two different structures. In one form (CCDC 2010272), the four-coordinate zinc atom binds to one nefiracetam molecule (*via* the γ-lactam carbonyl), one water molecule, and two chlorido ligands. In the second form (CCDC 2010264), the zinc bonds to one nefiracetam molecule through the γ-lactam and to a second *via* the amide carbonyl, forming a cyclic zinc dimer.

## Synthesis and crystallization   

All reagents were used as received from the manufacturer. Compounds (**1**) through (**5**) were prepared by dissolution of the respective components in various solvents [50:50 *v*:*v* ratio of water and ethanol (benzamide, 4-hy­droxy­benzamide), ethanol (*o*,*m*,*p*-tolu­amide)] followed by slow evaporation. In a typical preparation, a 1:1 stoichiometric ratio of benzamide (0.1352 g) and zinc(II) chloride (0.1336 g) was dissolved in approximately 5 mL of a 50:50 *v*:*v* ratio of water and ethanol. Slow evaporation of the resulting solution produced single crystals of compound (**1**).

## Refinement   

Crystal data, data collection and structure refinement details are summarized in Table 8 ▸. All hydrogen atoms were located in difference maps.

All carbon-bonded H atoms were placed in idealized positions using a riding model with aromatic C—H = 0.95 Å, methyl C—H = 0.98 Å and *U*_iso_(H) = 1.2*U*_eq_(C) (aromatic) or *U*_iso_(H) = 1.5*U*_eq_(C) (meth­yl). All amide H-atom positions were refined with N—H distances restrained to 0.88 (2) Å and U_iso_(H) = 1.5U_eq_(N). The hydroxyl H-atom positions in compound (**5**) were refined with O—H distances restrained to 0.84 (2) Å and *U*_iso_(H) = 1.5*U*_eq_(N).

Compound (**1**) was refined as a pseudo-merohedral twin (monoclinic mimicking ortho­rhom­bic, since β is close to 90°) with a twin law of (0 0 −1 0 −1 0 −1 0 0) , corresponding to a twofold rotation about the [10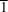] axis. The twin ratio refined to 0.4825 (5).

The methyl group in compound (**3**) was modeled as a disordered methyl group with each set of hydrogen atoms rotated by 60° (AFIX 127). The disorder was identified from multiple peaks near C8 in the difference map. The refined occupancies of the two hydrogen atom sets were 0.54 (2):0.46 (2).

## Supplementary Material

Crystal structure: contains datablock(s) global, 1, 2, 3, 4, 5. DOI: 10.1107/S2056989021008264/zl5023sup1.cif

Structure factors: contains datablock(s) 1. DOI: 10.1107/S2056989021008264/zl50231sup2.hkl

Supporting information file. DOI: 10.1107/S2056989021008264/zl50231sup7.mol

Structure factors: contains datablock(s) 2. DOI: 10.1107/S2056989021008264/zl50232sup3.hkl

Supporting information file. DOI: 10.1107/S2056989021008264/zl50232sup8.mol

Structure factors: contains datablock(s) 3. DOI: 10.1107/S2056989021008264/zl50233sup4.hkl

Supporting information file. DOI: 10.1107/S2056989021008264/zl50233sup9.mol

Supporting information file. DOI: 10.1107/S2056989021008264/zl50234sup10.mol

Structure factors: contains datablock(s) 4. DOI: 10.1107/S2056989021008264/zl50234sup5.hkl

Supporting information file. DOI: 10.1107/S2056989021008264/zl50235sup11.mol

Structure factors: contains datablock(s) 5. DOI: 10.1107/S2056989021008264/zl50235sup6.hkl

CCDC references: 2102513, 2102512, 2102511, 2102510, 2102509

Additional supporting information:  crystallographic information; 3D view; checkCIF report

## Figures and Tables

**Figure 5:**
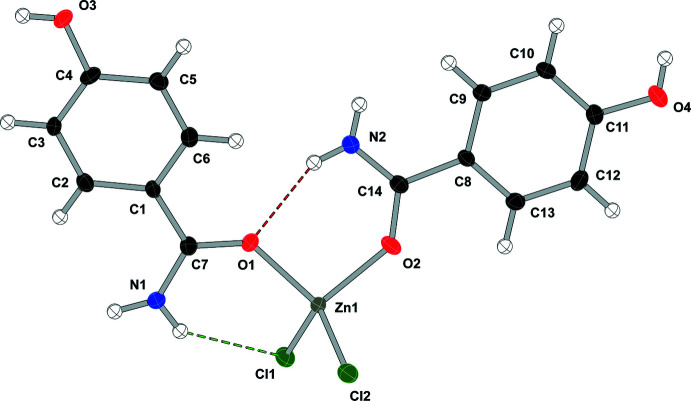
Displacement ellipsoid (50%) diagram and atom numbering scheme for (**5**). The N—H⋯O contact is shown in red and the N—H⋯Cl contact is shown in green.

**Table 1:** Hydrogen-bond geometry (Å, °) for (**1**)

*D*—H⋯*A*	*D*—H	H⋯*A*	*D*⋯*A*	*D*—H⋯*A*
N1*A*—H1*AA*⋯O2*A*	0.84 (2)	2.12 (2)	2.888 (2)	152 (3)
N1*A*—H1*AB*⋯Cl1*B* ^i^	0.87 (2)	2.56 (2)	3.3644 (15)	153 (2)
N2*A*—H2*AA*⋯Cl1*A*	0.87 (2)	2.51 (2)	3.3281 (15)	155 (2)
N2*A*—H2*AB*⋯Cl2*A* ^ii^	0.85 (2)	2.51 (2)	3.3404 (15)	164 (2)
N1*B*—H1*BA*⋯O2*B*	0.84 (2)	2.17 (2)	2.911 (2)	147 (2)
N1*B*—H1*BB*⋯Cl1*A*	0.88 (2)	2.51 (2)	3.3682 (16)	167 (2)
N2*B*—H2*BA*⋯Cl1*B*	0.85 (2)	2.57 (2)	3.3085 (15)	146 (2)
N2*B*—H2*BB*⋯Cl2*B* ^iii^	0.85 (2)	2.48 (2)	3.3107 (15)	165 (2)

Symmetry codes: (i) x-{\script{1\over 2}}, -y+{\script{3\over 2}}, z-{\script{1\over 2}}; (ii) -x+{\script{3\over 2}}, y+{\script{1\over 2}}, -z+{\script{1\over 2}}; (iii) -x+{\script{3\over 2}}, y-{\script{1\over 2}}, -z+{\script{3\over 2}}.

**Table 2:** Hydrogen-bond geometry (Å, °) for (**2**)

*D*—H⋯*A*	*D*—H	H⋯*A*	*D*⋯*A*	*D*—H⋯*A*
N1—H1*A*⋯Cl2^i^	0.82 (2)	2.57 (2)	3.2916 (17)	147 (2)
N1—H1*B*⋯Cl1	0.86 (2)	2.54 (2)	3.3077 (17)	150 (2)
N2—H2*A*⋯Cl1	0.85 (2)	2.52 (2)	3.2667 (16)	148 (2)
N2—H2*B*⋯O1^ii^	0.84 (2)	2.14 (2)	2.949 (2)	163 (2)

Symmetry codes: (i) x, y-1, z; (ii) x-1, y, z.

**Table 3:** Hydrogen-bond geometry (Å, °) for (**3**)

*D*—H⋯*A*	*D*—H	H⋯*A*	*D*⋯*A*	*D*—H⋯*A*
N1—H1*A*⋯Cl1^i^	0.85 (2)	2.56 (2)	3.2854 (13)	145 (2)
N1—H1*B*⋯Cl1^ii^	0.85 (2)	2.52 (2)	3.2979 (13)	153 (2)

Symmetry codes: (i) x, -y+1, z-{\script{1\over 2}}; (ii) x, y, z-1.

**Table 4:** Hydrogen-bond geometry (Å, °) for (**4**)

*D*—H⋯*A*	*D*—H	H⋯*A*	*D*⋯*A*	*D*—H⋯*A*
N1—H1*A*⋯O2	0.87 (2)	2.07 (2)	2.8753 (19)	154 (2)
N1—H1*B*⋯Cl2^i^	0.86 (2)	2.49 (2)	3.2265 (14)	145 (2)
N2—H2*A*⋯Cl1^ii^	0.86 (2)	2.50 (2)	3.2956 (16)	155 (2)
N2—H2*B*⋯Cl2	0.87 (2)	3.05 (2)	3.6341 (17)	126 (2)

Symmetry codes: (i) x, -y+{\script{1\over 2}}, z-{\script{1\over 2}}; (ii) x-1, y, z.

**Table 5:** Hydrogen-bond geometry (Å, °) for (**5**)

*D*—H⋯*A*	*D*—H	H⋯*A*	*D*⋯*A*	*D*—H⋯*A*
O3—H3⋯Cl1^i^	0.84 (3)	2.64 (4)	3.322 (3)	140 (5)
O3—H3⋯Cl2^ii^	0.84 (3)	2.75 (4)	3.349 (3)	130 (4)
O4—H4⋯Cl2^iii^	0.80 (3)	2.33 (3)	3.131 (3)	175 (6)
N1—H1*A*⋯Cl1	0.86 (3)	2.93 (4)	3.648 (4)	142 (4)
N1—H1*B*⋯Cl1^iv^	0.87 (3)	2.61 (3)	3.479 (4)	173 (4)
N2—H2*A*⋯O1	0.84 (3)	2.15 (3)	2.924 (5)	154 (5)
N2—H2*B*⋯Cl2^v^	0.84 (3)	2.77 (4)	3.405 (4)	135 (5)

Symmetry codes: (i) x, y, z+1; (ii) x+1, y, z+1; (iii) x+{\script{1\over 2}}, y-{\script{1\over 2}}, z; (iv) x, -y+2, z+{\script{1\over 2}}; (v) x+{\script{1\over 2}}, -y+{\script{3\over 2}}, z+{\script{1\over 2}}.

**Table 6:** Selected bond lengths and angles (Å, °) for compounds (**1**) through (**5**)

Compound	*R* / position	Zn—Cl1	Zn—Cl2	Zn—O1	Zn—O2	Cl—Zn—Cl	O—Zn—O
(**1**)*^*a*^*	H	2.2294 (4)	2.2118 (4)	1.9653 (12)	2.0040 (13)	113.726 (18)	99.75 (5)
(**1**)*^*b*^*	H	2.2361 (4)	2.2107 (4)	1.9632 (12)	2.0089 (13)	114.034 (18)	101.44 (5)
(**2**)	CH_3_ / *ortho*	2.2340 (4)	2.1947 (5)	2.0169 (13)	1.9781 (11)	125.120 (19)	103.92 (5)
(**3**)*^*c*^*	CH_3_ / *meta*	2.2341 (4)	2.2341 (4)	1.9652 (10)	1.9652 (10)	121.25 (2)	96.12 (6)
(**4**)	CH_3_ / *para*	2.2166 (5)	2.2170 (5)	1.9592 (12)	2.0191 (11)	115.836 (17)	101.98 (5)
(**5**)	OH / *para*	2.2347 (11)	2.2305 (11)	1.980 (3)	1.954 (3)	112.84 (4)	101.21 (12)

Notes: (*a*) mol­ecule 1; (*b*) mol­ecule 2; (*c*) O1/O2 and Cl1/Cl2 related by symmetry.

**Table 7:** Summary of π–π inter­actions (Å, °) in compounds (**1**), (**3**), and (**5**) α is the dihedral angle between planes. *Cg* is the centroid of the benzene ring of the benzamide or tolu­amide mol­ecule.

Compound	Ring *i*	Ring *j*	*Cg*⋯*Cg* distance	α
(**1**)	1	4^i^	3.9522 (11)	8.76 (9)
(**1**)	1	4^ii^	3.8781 (11)	8.76 (9)
(**1**)	3	2^iii^	3.8195 (10)	6.27 (8)
(**3**)	1	1^iv^	3.7770 (10)	6.86 (7)
(**5**)	1	2^v^	3.760 (3)	8.0 (2)

Symmetry codes: (i) 1 − *x*, 1 − *y*, 1 − *z*; (ii) 1 − *x*, 2 − *y*, 1 − *z*; (iii) {3\over 2} − *x*, −{1\over 2} + *y*, {1\over 2} − *z*; (iv) 1 − *x*, *y*, {1\over 2} − *z*; (v) −{1\over 2} + *x*, {3\over 2} − *y*, {1\over 2} + *z*.

**Table 8:** Experimental details

	(**1**)	(**2**)	(**3**)	(**4**)	(**5**)
Crystal data					
Chemical formula	[ZnCl_2_(C_7_H_7_NO)_2_]	[ZnCl_2_(C_8_H_9_NO)_2_]	[ZnCl_2_(C_8_H_9_NO)_2_]	[ZnCl_2_(C_8_H_9_NO)_2_]	[ZnCl_2_(C_7_H_7_NO_2_)_2_]
*M*_r_	378.54	406.59	406.59	406.59	410.54
Crystal system, space group	Monoclinic, *P*2_1_/*n*	Monoclinic, *P*2_1_	Monoclinic, *C*2/*c*	Monoclinic, *P*2_1_/*c*	Monoclinic, *C* *c*
Temperature (K)	100	100	100	100	100
*a*, *b*, *c* (Å)	20.6241 (11), 7.3309 (4), 20.6485 (11)	7.3802 (3), 8.2491 (3), 14.5953 (5)	13.9452 (11), 18.9742 (16), 7.0651 (6)	6.8376 (4), 17.2694 (9), 14.9856 (7)	7.0532 (6), 21.3776 (17), 11.1181 (9)
β (°)	90.532 (1)	97.852 (1)	108.021 (2)	96.893 (2)	106.477 (2)
*V* (Å^3^)	3121.8 (3)	880.23 (6)	1777.7 (3)	1756.73 (16)	1607.5 (2)
*Z*	8	2	4	4	4
Radiation type	Mo *K*α	Mo *K*α	Mo *K*α	Mo *K*α	Mo *K*α
μ (mm^−1^)	1.92	1.71	1.69	1.71	1.88
Crystal size (mm)	0.6 × 0.60 × 0.35	0.5 × 0.16 × 0.11	0.42 × 0.14 × 0.14	0.56 × 0.18 × 0.09	0.15 × 0.09 × 0.07

Data collection					
Diffractometer	Bruker APEXII CCD	Bruker APEXII CCD	Bruker APEXII CCD	Bruker APEXII CCD	Bruker APEXII CCD
Absorption correction	Multi-scan (*SADABS*; Krause *et al.*, 2015 ▸)	Multi-scan (*SADABS*; Krause *et al.*, 2015 ▸)	Multi-scan (*SADABS*; Krause *et al.*, 2015 ▸)	Multi-scan (*SADABS*; Krause *et al.*, 2015 ▸)	Multi-scan (*SADABS*; Krause *et al.*, 2015 ▸)
*T*_min_, *T*_max_	0.558, 0.746	0.478, 0.680	0.620, 0.746	0.629, 0.746	0.673, 0.746
No. of measured, independent and observed [*I* > 2σ(*I*)] reflections	48491, 9668, 9501	20749, 5348, 5135	12177, 2295, 2023	33806, 5376, 4283	17255, 4168, 3809
*R*_int_	0.023	0.025	0.027	0.051	0.042
(sin θ/λ)_max_ (Å^−1^)	0.718	0.714	0.676	0.715	0.676

Refinement					
*R*[*F*^2^ > 2σ(*F* ^2^)], *wR*(*F* ^2^), *S*	0.022, 0.053, 1.07	0.018, 0.039, 1.00	0.022, 0.059, 1.05	0.031, 0.069, 1.01	0.030, 0.065, 1.05
No. of reflections	9668	5348	2295	5376	4168
No. of parameters	404	223	113	222	227
No. of restraints	8	5	17	4	8
H-atom treatment	H atoms treated by a mixture of independent and constrained refinement	H atoms treated by a mixture of independent and constrained refinement	H atoms treated by a mixture of independent and constrained refinement	H atoms treated by a mixture of independent and constrained refinement	H atoms treated by a mixture of independent and constrained refinement
Δρ_max_, Δρ_min_ (e Å^−3^)	0.43, −0.35	0.31, −0.24	0.39, −0.26	0.46, −0.32	0.46, −0.29
Absolute structure	–	Refined as an inversion twin.	–	–	Refined as an inversion twin
Absolute structure parameter	–	0.016 (6)	–	–	0.024 (13)

Computer programs: *BIS* (Bruker, 2020 ▸), *SAINT* (Bruker, 2020 ▸), *SHELXT* (Sheldrick, 2015*a* ▸), *SHELXL2018/3* (Sheldrick, 2015*b* ▸), *CrystalMaker* (Palmer, 2020 ▸), *OLEX2* (Dolomanov *et al.*, 2009 ▸), and *publCIF* (Westrip, 2010 ▸).

## supplementary crystallographic information

### Bis(benzamide-κO)dichloridozinc(II) (1) Crystal data

**Table:**

[ZnCl_2_(C_7_H_7_NO)_2_]	*F*(000) = 1536
*M**_r_* = 378.54	*D*_x_ = 1.611 Mg m^−^^3^
Monoclinic, *P*2_1_/*n*	Mo *K*α radiation, λ = 0.71073 Å
*a* = 20.6241 (11) Å	Cell parameters from 9702 reflections
*b* = 7.3309 (4) Å	θ = 6.8–30.5°
*c* = 20.6485 (11) Å	µ = 1.92 mm^−^^1^
β = 90.532 (1)°	*T* = 100 K
*V* = 3121.8 (3) Å^3^	Block, clear light colourless
*Z* = 8	0.6 × 0.60 × 0.35 mm

### Bis(benzamide-κO)dichloridozinc(II) (1) Data collection

**Table:**

Bruker APEXII CCD diffractometer	9668 independent reflections
Radiation source: sealed tube	9501 reflections with *I* > 2σ(*I*)
Graphite monochromator	*R*_int_ = 0.023
Detector resolution: 8 pixels mm^-1^	θ_max_ = 30.7°, θ_min_ = 1.0°
ω and φ scans	*h* = −29→27
Absorption correction: multi-scan (SADABS; Krause *et al.*, 2015)	*k* = −10→10
*T*_min_ = 0.558, *T*_max_ = 0.746	*l* = −29→29
48491 measured reflections	

### Bis(benzamide-κO)dichloridozinc(II) (1) Refinement

**Table:**

Refinement on *F*^2^	Primary atom site location: dual
Least-squares matrix: full	Hydrogen site location: mixed
*R*[*F*^2^ > 2σ(*F*^2^)] = 0.022	H atoms treated by a mixture of independent and constrained refinement
*wR*(*F*^2^) = 0.053	*w* = 1/[σ^2^(*F*_o_^2^) + (0.0256*P*)^2^ + 1.2394*P*] where *P* = (*F*_o_^2^ + 2*F*_c_^2^)/3
*S* = 1.07	(Δ/σ)_max_ = 0.002
9668 reflections	Δρ_max_ = 0.43 e Å^−^^3^
404 parameters	Δρ_min_ = −0.34 e Å^−^^3^
8 restraints	

### Bis(benzamide-κO)dichloridozinc(II) (1) Special details

**Table:**

Geometry. All esds (except the esd in the dihedral angle between two l.s. planes) are estimated using the full covariance matrix. The cell esds are taken into account individually in the estimation of esds in distances, angles and torsion angles; correlations between esds in cell parameters are only used when they are defined by crystal symmetry. An approximate (isotropic) treatment of cell esds is used for estimating esds involving l.s. planes.
Refinement. Refined as a 2-component twin.

### Bis(benzamide-κO)dichloridozinc(II) (1) Fractional atomic coordinates and isotropic or equivalent isotropic displacement parameters (Å^2^)

**Table:**

	*x*	*y*	*z*	*U*_iso_*/*U*_eq_	
Zn1A	0.62741 (2)	0.74478 (4)	0.34018 (2)	0.01179 (4)	
Cl1A	0.69397 (2)	0.97842 (5)	0.36146 (2)	0.01636 (7)	
Cl2A	0.66272 (2)	0.47923 (6)	0.37699 (2)	0.02204 (9)	
O1A	0.53865 (6)	0.78998 (17)	0.36973 (6)	0.0163 (2)	
O2A	0.61465 (6)	0.73870 (17)	0.24363 (6)	0.0163 (2)	
N1A	0.48113 (7)	0.8093 (2)	0.27621 (8)	0.0217 (3)	
H1AA	0.5138 (10)	0.785 (4)	0.2536 (12)	0.033*	
H1AB	0.4458 (10)	0.824 (3)	0.2534 (11)	0.033*	
N2A	0.71341 (7)	0.8269 (2)	0.21095 (7)	0.0189 (3)	
H2AA	0.7218 (12)	0.869 (3)	0.2497 (9)	0.028*	
H2AB	0.7417 (10)	0.851 (3)	0.1825 (10)	0.028*	
C1A	0.42560 (7)	0.8246 (2)	0.37895 (8)	0.0138 (3)	
C2A	0.36411 (9)	0.7932 (2)	0.35252 (10)	0.0188 (3)	
H2A	0.359316	0.761280	0.308143	0.023*	
C3A	0.30976 (9)	0.8092 (3)	0.39175 (11)	0.0251 (4)	
H3A	0.267689	0.788739	0.374069	0.030*	
C4A	0.31709 (9)	0.8548 (3)	0.45663 (10)	0.0257 (4)	
H4A	0.279853	0.865296	0.483113	0.031*	
C5A	0.37808 (9)	0.8854 (3)	0.48331 (9)	0.0245 (4)	
H5A	0.382768	0.916605	0.527769	0.029*	
C6A	0.43205 (8)	0.8700 (2)	0.44430 (8)	0.0188 (3)	
H6A	0.473993	0.890556	0.462261	0.023*	
C7A	0.48516 (7)	0.8061 (2)	0.34012 (8)	0.0137 (3)	
C8A	0.63351 (8)	0.7328 (2)	0.13053 (8)	0.0129 (3)	
C9A	0.67309 (8)	0.7703 (2)	0.07743 (8)	0.0156 (3)	
H9A	0.715796	0.815424	0.084231	0.019*	
C10A	0.64995 (9)	0.7416 (2)	0.01464 (9)	0.0190 (3)	
H10A	0.676912	0.767083	−0.021295	0.023*	
C11A	0.58777 (9)	0.6760 (2)	0.00452 (8)	0.0201 (3)	
H11A	0.572142	0.656193	−0.038327	0.024*	
C12A	0.54825 (8)	0.6391 (2)	0.05687 (8)	0.0201 (3)	
H12A	0.505440	0.595321	0.049643	0.024*	
C13A	0.57073 (8)	0.6657 (2)	0.11984 (8)	0.0163 (3)	
H13A	0.543606	0.638474	0.155497	0.020*	
C14A	0.65448 (8)	0.7671 (2)	0.19832 (8)	0.0129 (3)	
Zn1B	0.83932 (2)	0.71554 (2)	0.62742 (2)	0.01256 (4)	
Cl1B	0.86157 (2)	0.48139 (6)	0.69283 (2)	0.01790 (8)	
Cl2B	0.87722 (2)	0.97969 (6)	0.66307 (2)	0.02414 (8)	
O1B	0.86683 (6)	0.67828 (17)	0.53751 (6)	0.0176 (2)	
O2B	0.74288 (6)	0.71225 (17)	0.61469 (6)	0.0171 (2)	
N1B	0.77974 (8)	0.7747 (2)	0.48074 (8)	0.0190 (3)	
H1BA	0.7583 (12)	0.791 (3)	0.5147 (10)	0.028*	
H1BB	0.7610 (11)	0.815 (3)	0.4453 (9)	0.028*	
N2B	0.71169 (7)	0.6279 (2)	0.71506 (7)	0.0201 (3)	
H2BA	0.7511 (8)	0.612 (3)	0.7260 (11)	0.030*	
H2BB	0.6836 (10)	0.585 (3)	0.7407 (10)	0.030*	
C1B	0.87943 (7)	0.7032 (2)	0.42412 (8)	0.0134 (3)	
C2B	0.85410 (10)	0.7395 (2)	0.36242 (9)	0.0191 (3)	
H2B	0.810046	0.775075	0.357395	0.023*	
C3B	0.89345 (10)	0.7234 (3)	0.30844 (9)	0.0231 (4)	
H3B	0.876266	0.747726	0.266478	0.028*	
C4B	0.95793 (9)	0.6716 (3)	0.31582 (9)	0.0229 (3)	
H4B	0.984945	0.661451	0.278984	0.028*	
C5B	0.98268 (8)	0.6349 (2)	0.37696 (9)	0.0221 (3)	
H5B	1.026722	0.599079	0.381792	0.027*	
C6B	0.94395 (7)	0.6499 (2)	0.43130 (8)	0.0165 (3)	
H6B	0.961289	0.623994	0.473079	0.020*	
C7B	0.84054 (8)	0.7192 (2)	0.48449 (8)	0.0133 (3)	
C8B	0.63040 (7)	0.6729 (2)	0.63094 (8)	0.0135 (3)	
C9B	0.57871 (8)	0.6850 (2)	0.67379 (8)	0.0176 (3)	
H9B	0.586500	0.697499	0.718986	0.021*	
C10B	0.51568 (8)	0.6786 (3)	0.64956 (8)	0.0189 (3)	
H10B	0.480096	0.687201	0.678286	0.023*	
C11B	0.50456 (8)	0.6597 (2)	0.58355 (8)	0.0196 (3)	
H11B	0.461330	0.655944	0.567321	0.024*	
C12B	0.55602 (8)	0.6463 (3)	0.54103 (8)	0.0197 (3)	
H12B	0.548141	0.632735	0.495882	0.024*	
C13B	0.61889 (8)	0.6528 (2)	0.56485 (8)	0.0164 (3)	
H13B	0.654323	0.643623	0.535959	0.020*	
C14B	0.69873 (8)	0.6727 (2)	0.65418 (8)	0.0151 (3)	

### Bis(benzamide-κO)dichloridozinc(II) (1) Atomic displacement parameters (Å^2^)

**Table:**

	*U*^11^	*U*^22^	*U*^33^	*U*^12^	*U*^13^	*U*^23^
Zn1A	0.00847 (10)	0.01580 (8)	0.01112 (10)	0.00076 (6)	0.00080 (5)	0.00048 (6)
Cl1A	0.01556 (17)	0.01763 (16)	0.01590 (17)	−0.00279 (13)	0.00038 (13)	−0.00214 (13)
Cl2A	0.01553 (18)	0.02015 (18)	0.0306 (2)	0.00577 (13)	0.00699 (15)	0.00859 (15)
O1A	0.0097 (5)	0.0253 (6)	0.0139 (5)	0.0034 (4)	−0.0001 (4)	−0.0007 (4)
O2A	0.0124 (6)	0.0253 (6)	0.0112 (6)	−0.0021 (4)	0.0016 (4)	−0.0004 (4)
N1A	0.0101 (6)	0.0408 (9)	0.0143 (7)	0.0017 (6)	0.0003 (5)	0.0039 (6)
N2A	0.0108 (6)	0.0342 (8)	0.0118 (6)	−0.0019 (5)	0.0012 (4)	−0.0034 (5)
C1A	0.0101 (6)	0.0145 (7)	0.0169 (7)	0.0014 (5)	0.0035 (5)	0.0027 (5)
C2A	0.0098 (8)	0.0244 (8)	0.0221 (9)	−0.0006 (6)	0.0017 (6)	0.0051 (7)
C3A	0.0095 (7)	0.0296 (9)	0.0362 (11)	0.0009 (6)	0.0040 (7)	0.0075 (8)
C4A	0.0174 (8)	0.0252 (9)	0.0348 (10)	0.0037 (7)	0.0140 (7)	0.0029 (7)
C5A	0.0230 (8)	0.0269 (9)	0.0238 (8)	0.0021 (7)	0.0106 (6)	−0.0049 (7)
C6A	0.0143 (7)	0.0217 (8)	0.0206 (8)	0.0018 (6)	0.0041 (6)	−0.0027 (6)
C7A	0.0095 (6)	0.0150 (7)	0.0166 (7)	0.0007 (5)	0.0020 (5)	0.0014 (5)
C8A	0.0122 (7)	0.0160 (7)	0.0104 (6)	0.0026 (5)	−0.0005 (5)	−0.0011 (5)
C9A	0.0127 (7)	0.0215 (8)	0.0125 (7)	0.0033 (5)	0.0017 (5)	−0.0002 (5)
C10A	0.0198 (8)	0.0233 (8)	0.0140 (7)	0.0062 (6)	0.0012 (6)	0.0000 (6)
C11A	0.0231 (8)	0.0223 (8)	0.0149 (7)	0.0043 (6)	−0.0027 (6)	−0.0039 (6)
C12A	0.0189 (7)	0.0218 (8)	0.0194 (8)	−0.0022 (6)	−0.0035 (6)	−0.0047 (6)
C13A	0.0165 (7)	0.0178 (7)	0.0146 (7)	−0.0012 (6)	0.0006 (5)	−0.0022 (5)
C14A	0.0108 (7)	0.0166 (7)	0.0114 (7)	0.0024 (5)	−0.0002 (5)	0.0000 (5)
Zn1B	0.01035 (10)	0.01706 (8)	0.01029 (10)	0.00033 (6)	0.00080 (6)	−0.00034 (6)
Cl1B	0.01485 (17)	0.01905 (17)	0.01981 (19)	0.00226 (12)	−0.00040 (14)	0.00404 (13)
Cl2B	0.0299 (2)	0.02135 (17)	0.0213 (2)	−0.00772 (15)	0.00810 (15)	−0.00662 (14)
O1B	0.0148 (5)	0.0277 (6)	0.0103 (5)	0.0031 (5)	0.0004 (4)	−0.0016 (4)
O2B	0.0104 (6)	0.0269 (6)	0.0140 (6)	0.0009 (4)	0.0028 (4)	0.0032 (4)
N1B	0.0126 (7)	0.0326 (8)	0.0117 (7)	0.0026 (5)	0.0015 (5)	0.0016 (5)
N2B	0.0114 (6)	0.0358 (8)	0.0130 (6)	0.0005 (6)	0.0010 (5)	0.0033 (6)
C1B	0.0126 (7)	0.0150 (7)	0.0126 (7)	−0.0014 (5)	0.0023 (5)	−0.0019 (5)
C2B	0.0194 (9)	0.0240 (8)	0.0139 (8)	0.0010 (6)	0.0000 (6)	0.0001 (6)
C3B	0.0279 (10)	0.0283 (9)	0.0133 (8)	0.0025 (7)	0.0047 (7)	0.0015 (6)
C4B	0.0282 (9)	0.0234 (8)	0.0173 (8)	0.0013 (7)	0.0108 (6)	−0.0004 (6)
C5B	0.0186 (8)	0.0254 (8)	0.0225 (8)	0.0015 (6)	0.0077 (6)	−0.0033 (6)
C6B	0.0133 (7)	0.0197 (7)	0.0164 (7)	0.0011 (6)	0.0030 (5)	−0.0017 (6)
C7B	0.0120 (7)	0.0158 (7)	0.0120 (7)	−0.0023 (5)	0.0016 (5)	−0.0029 (5)
C8B	0.0108 (6)	0.0152 (7)	0.0146 (7)	−0.0008 (5)	0.0002 (5)	−0.0013 (5)
C9B	0.0140 (7)	0.0224 (8)	0.0163 (8)	0.0008 (6)	0.0013 (6)	−0.0035 (6)
C10B	0.0108 (7)	0.0268 (8)	0.0191 (8)	−0.0014 (6)	0.0032 (5)	−0.0036 (6)
C11B	0.0120 (7)	0.0252 (8)	0.0216 (8)	−0.0039 (6)	−0.0030 (6)	−0.0002 (6)
C12B	0.0166 (7)	0.0282 (9)	0.0142 (7)	−0.0051 (6)	−0.0021 (5)	0.0003 (6)
C13B	0.0143 (7)	0.0198 (7)	0.0151 (7)	−0.0029 (6)	0.0011 (5)	0.0008 (6)
C14B	0.0129 (7)	0.0176 (7)	0.0147 (7)	0.0009 (6)	0.0023 (5)	−0.0015 (6)

### Bis(benzamide-κO)dichloridozinc(II) (1) Geometric parameters (Å, º)

**Table:**

Zn1A—Cl1A	2.2361 (4)	Zn1B—Cl1B	2.2294 (4)
Zn1A—Cl2A	2.2107 (4)	Zn1B—Cl2B	2.2118 (4)
Zn1A—O1A	1.9632 (12)	Zn1B—O1B	1.9653 (12)
Zn1A—O2A	2.0089 (13)	Zn1B—O2B	2.0040 (13)
O1A—C7A	1.2618 (19)	O1B—C7B	1.254 (2)
O2A—C14A	1.268 (2)	O2B—C14B	1.2617 (19)
N1A—H1AA	0.842 (16)	N1B—H1BA	0.842 (16)
N1A—H1AB	0.871 (16)	N1B—H1BB	0.876 (16)
N1A—C7A	1.322 (2)	N1B—C7B	1.320 (2)
N2A—H2AA	0.874 (16)	N2B—H2BA	0.850 (16)
N2A—H2AB	0.851 (16)	N2B—H2BB	0.848 (16)
N2A—C14A	1.316 (2)	N2B—C14B	1.324 (2)
C1A—C2A	1.395 (2)	C1B—C2B	1.398 (2)
C1A—C6A	1.395 (2)	C1B—C6B	1.393 (2)
C1A—C7A	1.479 (2)	C1B—C7B	1.493 (2)
C2A—H2A	0.9500	C2B—H2B	0.9500
C2A—C3A	1.394 (3)	C2B—C3B	1.390 (3)
C3A—H3A	0.9500	C3B—H3B	0.9500
C3A—C4A	1.388 (3)	C3B—C4B	1.390 (3)
C4A—H4A	0.9500	C4B—H4B	0.9500
C4A—C5A	1.387 (3)	C4B—C5B	1.384 (3)
C5A—H5A	0.9500	C5B—H5B	0.9500
C5A—C6A	1.385 (2)	C5B—C6B	1.388 (2)
C6A—H6A	0.9500	C6B—H6B	0.9500
C8A—C9A	1.400 (2)	C8B—C9B	1.394 (2)
C8A—C13A	1.401 (2)	C8B—C13B	1.391 (2)
C8A—C14A	1.483 (2)	C8B—C14B	1.485 (2)
C9A—H9A	0.9500	C9B—H9B	0.9500
C9A—C10A	1.394 (2)	C9B—C10B	1.389 (2)
C10A—H10A	0.9500	C10B—H10B	0.9500
C10A—C11A	1.384 (3)	C10B—C11B	1.387 (2)
C11A—H11A	0.9500	C11B—H11B	0.9500
C11A—C12A	1.386 (2)	C11B—C12B	1.387 (2)
C12A—H12A	0.9500	C12B—H12B	0.9500
C12A—C13A	1.390 (2)	C12B—C13B	1.383 (2)
C13A—H13A	0.9500	C13B—H13B	0.9500
			
Cl2A—Zn1A—Cl1A	114.035 (18)	Cl2B—Zn1B—Cl1B	113.726 (18)
O1A—Zn1A—Cl1A	112.47 (4)	O1B—Zn1B—Cl1B	113.93 (4)
O1A—Zn1A—Cl2A	110.29 (4)	O1B—Zn1B—Cl2B	109.38 (4)
O1A—Zn1A—O2A	101.44 (5)	O1B—Zn1B—O2B	99.75 (5)
O2A—Zn1A—Cl1A	106.65 (4)	O2B—Zn1B—Cl1B	105.59 (4)
O2A—Zn1A—Cl2A	111.18 (4)	O2B—Zn1B—Cl2B	113.67 (4)
C7A—O1A—Zn1A	132.74 (11)	C7B—O1B—Zn1B	131.70 (11)
C14A—O2A—Zn1A	130.46 (11)	C14B—O2B—Zn1B	129.74 (12)
H1AA—N1A—H1AB	113 (3)	H1BA—N1B—H1BB	115 (3)
C7A—N1A—H1AA	120 (2)	C7B—N1B—H1BA	120.1 (18)
C7A—N1A—H1AB	125.9 (18)	C7B—N1B—H1BB	124.4 (17)
H2AA—N2A—H2AB	115 (2)	H2BA—N2B—H2BB	116 (2)
C14A—N2A—H2AA	118.3 (16)	C14B—N2B—H2BA	118.1 (17)
C14A—N2A—H2AB	124.9 (16)	C14B—N2B—H2BB	123.5 (16)
C2A—C1A—C7A	121.96 (15)	C2B—C1B—C7B	123.15 (15)
C6A—C1A—C2A	119.73 (15)	C6B—C1B—C2B	119.91 (15)
C6A—C1A—C7A	118.29 (14)	C6B—C1B—C7B	116.94 (14)
C1A—C2A—H2A	120.3	C1B—C2B—H2B	120.0
C3A—C2A—C1A	119.46 (19)	C3B—C2B—C1B	119.91 (18)
C3A—C2A—H2A	120.3	C3B—C2B—H2B	120.0
C2A—C3A—H3A	120.0	C2B—C3B—H3B	120.0
C4A—C3A—C2A	120.01 (18)	C2B—C3B—C4B	120.03 (18)
C4A—C3A—H3A	120.0	C4B—C3B—H3B	120.0
C3A—C4A—H4A	119.6	C3B—C4B—H4B	120.1
C5A—C4A—C3A	120.86 (16)	C5B—C4B—C3B	119.84 (16)
C5A—C4A—H4A	119.6	C5B—C4B—H4B	120.1
C4A—C5A—H5A	120.4	C4B—C5B—H5B	119.6
C6A—C5A—C4A	119.12 (18)	C4B—C5B—C6B	120.80 (16)
C6A—C5A—H5A	120.4	C6B—C5B—H5B	119.6
C1A—C6A—H6A	119.6	C1B—C6B—H6B	120.3
C5A—C6A—C1A	120.81 (16)	C5B—C6B—C1B	119.50 (16)
C5A—C6A—H6A	119.6	C5B—C6B—H6B	120.3
O1A—C7A—N1A	122.15 (15)	O1B—C7B—N1B	121.88 (15)
O1A—C7A—C1A	118.20 (15)	O1B—C7B—C1B	118.61 (14)
N1A—C7A—C1A	119.64 (14)	N1B—C7B—C1B	119.51 (15)
C9A—C8A—C13A	119.37 (15)	C9B—C8B—C14B	121.61 (14)
C9A—C8A—C14A	122.60 (15)	C13B—C8B—C9B	120.32 (15)
C13A—C8A—C14A	117.99 (14)	C13B—C8B—C14B	118.02 (14)
C8A—C9A—H9A	119.9	C8B—C9B—H9B	120.4
C10A—C9A—C8A	120.11 (16)	C10B—C9B—C8B	119.19 (16)
C10A—C9A—H9A	119.9	C10B—C9B—H9B	120.4
C9A—C10A—H10A	119.9	C9B—C10B—H10B	119.9
C11A—C10A—C9A	120.13 (17)	C11B—C10B—C9B	120.19 (16)
C11A—C10A—H10A	119.9	C11B—C10B—H10B	119.9
C10A—C11A—H11A	120.0	C10B—C11B—H11B	119.7
C10A—C11A—C12A	120.05 (16)	C10B—C11B—C12B	120.57 (15)
C12A—C11A—H11A	120.0	C12B—C11B—H11B	119.7
C11A—C12A—H12A	119.7	C11B—C12B—H12B	120.2
C11A—C12A—C13A	120.57 (16)	C13B—C12B—C11B	119.51 (16)
C13A—C12A—H12A	119.7	C13B—C12B—H12B	120.2
C8A—C13A—H13A	120.1	C8B—C13B—H13B	119.9
C12A—C13A—C8A	119.76 (15)	C12B—C13B—C8B	120.22 (15)
C12A—C13A—H13A	120.1	C12B—C13B—H13B	119.9
O2A—C14A—N2A	120.79 (15)	O2B—C14B—N2B	122.02 (15)
O2A—C14A—C8A	118.92 (15)	O2B—C14B—C8B	118.66 (15)
N2A—C14A—C8A	120.29 (14)	N2B—C14B—C8B	119.31 (14)
			
Zn1A—O1A—C7A—N1A	−6.1 (2)	Zn1B—O1B—C7B—N1B	−11.6 (2)
Zn1A—O1A—C7A—C1A	174.83 (11)	Zn1B—O1B—C7B—C1B	168.80 (11)
Zn1A—O2A—C14A—N2A	−7.2 (2)	Zn1B—O2B—C14B—N2B	1.6 (3)
Zn1A—O2A—C14A—C8A	173.10 (10)	Zn1B—O2B—C14B—C8B	−177.09 (11)
C1A—C2A—C3A—C4A	−0.4 (3)	C1B—C2B—C3B—C4B	−0.1 (3)
C2A—C1A—C6A—C5A	−0.4 (3)	C2B—C1B—C6B—C5B	0.6 (2)
C2A—C1A—C7A—O1A	−162.09 (15)	C2B—C1B—C7B—O1B	177.76 (15)
C2A—C1A—C7A—N1A	18.8 (2)	C2B—C1B—C7B—N1B	−1.9 (2)
C2A—C3A—C4A—C5A	0.1 (3)	C2B—C3B—C4B—C5B	0.5 (3)
C3A—C4A—C5A—C6A	0.0 (3)	C3B—C4B—C5B—C6B	−0.3 (3)
C4A—C5A—C6A—C1A	0.1 (3)	C4B—C5B—C6B—C1B	−0.3 (3)
C6A—C1A—C2A—C3A	0.5 (3)	C6B—C1B—C2B—C3B	−0.4 (3)
C6A—C1A—C7A—O1A	16.1 (2)	C6B—C1B—C7B—O1B	−2.4 (2)
C6A—C1A—C7A—N1A	−162.95 (16)	C6B—C1B—C7B—N1B	177.91 (15)
C7A—C1A—C2A—C3A	178.72 (16)	C7B—C1B—C2B—C3B	179.38 (16)
C7A—C1A—C6A—C5A	−178.66 (16)	C7B—C1B—C6B—C5B	−179.20 (15)
C8A—C9A—C10A—C11A	0.0 (2)	C8B—C9B—C10B—C11B	0.2 (3)
C9A—C8A—C13A—C12A	−0.7 (2)	C9B—C8B—C13B—C12B	0.5 (3)
C9A—C8A—C14A—O2A	176.29 (15)	C9B—C8B—C14B—O2B	−160.50 (16)
C9A—C8A—C14A—N2A	−3.4 (2)	C9B—C8B—C14B—N2B	20.8 (3)
C9A—C10A—C11A—C12A	0.2 (3)	C9B—C10B—C11B—C12B	0.2 (3)
C10A—C11A—C12A—C13A	−0.7 (3)	C10B—C11B—C12B—C13B	−0.3 (3)
C11A—C12A—C13A—C8A	0.9 (3)	C11B—C12B—C13B—C8B	0.0 (3)
C13A—C8A—C9A—C10A	0.2 (2)	C13B—C8B—C9B—C10B	−0.6 (3)
C13A—C8A—C14A—O2A	−1.6 (2)	C13B—C8B—C14B—O2B	22.1 (2)
C13A—C8A—C14A—N2A	178.73 (15)	C13B—C8B—C14B—N2B	−156.62 (16)
C14A—C8A—C9A—C10A	−177.62 (15)	C14B—C8B—C9B—C10B	−177.92 (16)
C14A—C8A—C13A—C12A	177.25 (15)	C14B—C8B—C13B—C12B	177.94 (16)

### Bis(benzamide-κO)dichloridozinc(II) (1) Hydrogen-bond geometry (Å, º)

**Table:**

*D*—H···*A*	*D*—H	H···*A*	*D*···*A*	*D*—H···*A*
N1*A*—H1*AA*···O2*A*	0.84 (2)	2.12 (2)	2.888 (2)	152 (3)
N1*A*—H1*AB*···Cl1*B*^i^	0.87 (2)	2.56 (2)	3.3644 (15)	153 (2)
N2*A*—H2*AA*···Cl1*A*	0.87 (2)	2.51 (2)	3.3281 (15)	155 (2)
N2*A*—H2*AB*···Cl2*A*^ii^	0.85 (2)	2.51 (2)	3.3404 (15)	164 (2)
N1*B*—H1*BA*···O2*B*	0.84 (2)	2.17 (2)	2.911 (2)	147 (2)
N1*B*—H1*BB*···Cl1*A*	0.88 (2)	2.51 (2)	3.3682 (16)	167 (2)
N2*B*—H2*BA*···Cl1*B*	0.85 (2)	2.57 (2)	3.3085 (15)	146 (2)
N2*B*—H2*BB*···Cl2*B*^iii^	0.85 (2)	2.48 (2)	3.3107 (15)	165 (2)

Symmetry codes: (i) *x*−1/2, −*y*+3/2, *z*−1/2; (ii) −*x*+3/2, *y*+1/2, −*z*+1/2; (iii) −*x*+3/2, *y*−1/2, −*z*+3/2.

### Dichloridobis(2-methylbenzamide-κO)zinc(II) (2) Crystal data

**Table:**

[ZnCl_2_(C_8_H_9_NO)_2_]	*F*(000) = 416
*M**_r_* = 406.59	*D*_x_ = 1.534 Mg m^−^^3^
Monoclinic, *P*2_1_	Mo *K*α radiation, λ = 0.71073 Å
*a* = 7.3802 (3) Å	Cell parameters from 9884 reflections
*b* = 8.2491 (3) Å	θ = 2.8–30.5°
*c* = 14.5953 (5) Å	µ = 1.71 mm^−^^1^
β = 97.852 (1)°	*T* = 100 K
*V* = 880.23 (6) Å^3^	Needle, clear light colourless
*Z* = 2	0.5 × 0.16 × 0.11 mm

### Dichloridobis(2-methylbenzamide-κO)zinc(II) (2) Data collection

**Table:**

Bruker APEXII CCD diffractometer	5348 independent reflections
Radiation source: sealed tube	5135 reflections with *I* > 2σ(*I*)
Graphite monochromator	*R*_int_ = 0.025
Detector resolution: 8 pixels mm^-1^	θ_max_ = 30.5°, θ_min_ = 2.8°
ω and φ scans	*h* = −10→10
Absorption correction: multi-scan (SADABS; Krause *et al.*, 2015)	*k* = −11→11
*T*_min_ = 0.478, *T*_max_ = 0.680	*l* = −20→20
20749 measured reflections	

### Dichloridobis(2-methylbenzamide-κO)zinc(II) (2) Refinement

**Table:**

Refinement on *F*^2^	Hydrogen site location: mixed
Least-squares matrix: full	H atoms treated by a mixture of independent and constrained refinement
*R*[*F*^2^ > 2σ(*F*^2^)] = 0.018	*w* = 1/[σ^2^(*F*_o_^2^) + (0.0078*P*)^2^] where *P* = (*F*_o_^2^ + 2*F*_c_^2^)/3
*wR*(*F*^2^) = 0.039	(Δ/σ)_max_ < 0.001
*S* = 1.00	Δρ_max_ = 0.31 e Å^−^^3^
5348 reflections	Δρ_min_ = −0.24 e Å^−^^3^
223 parameters	Absolute structure: Refined as an inversion twin.
5 restraints	Absolute structure parameter: 0.016 (6)
Primary atom site location: dual	

### Dichloridobis(2-methylbenzamide-κO)zinc(II) (2) Special details

**Table:**

Geometry. All esds (except the esd in the dihedral angle between two l.s. planes) are estimated using the full covariance matrix. The cell esds are taken into account individually in the estimation of esds in distances, angles and torsion angles; correlations between esds in cell parameters are only used when they are defined by crystal symmetry. An approximate (isotropic) treatment of cell esds is used for estimating esds involving l.s. planes.
Refinement. Refined as a 2-component inversion twin

### Dichloridobis(2-methylbenzamide-κO)zinc(II) (2) Fractional atomic coordinates and isotropic or equivalent isotropic displacement parameters (Å^2^)

**Table:**

	*x*	*y*	*z*	*U*_iso_*/*U*_eq_	
Zn1	0.65448 (2)	0.68146 (2)	0.17755 (2)	0.01057 (5)	
Cl1	0.47230 (6)	0.60339 (6)	0.05028 (3)	0.01680 (9)	
Cl2	0.81306 (6)	0.90729 (5)	0.19165 (3)	0.01797 (10)	
O1	0.85119 (17)	0.51161 (15)	0.20425 (9)	0.0127 (2)	
O2	0.51677 (14)	0.66731 (18)	0.28443 (8)	0.0146 (2)	
N1	0.7092 (2)	0.28760 (19)	0.14025 (13)	0.0191 (4)	
H1A	0.719 (3)	0.190 (3)	0.1287 (15)	0.029*	
H1B	0.616 (3)	0.340 (3)	0.1134 (17)	0.029*	
N2	0.2228 (2)	0.64887 (18)	0.21691 (10)	0.0143 (3)	
H2A	0.253 (3)	0.665 (3)	0.1637 (13)	0.021*	
H2B	0.114 (3)	0.627 (3)	0.2209 (15)	0.021*	
C1	1.0259 (2)	0.2690 (2)	0.20486 (12)	0.0104 (3)	
C2	1.1130 (2)	0.1966 (2)	0.13538 (11)	0.0133 (3)	
C3	1.2723 (2)	0.1092 (2)	0.16300 (13)	0.0169 (4)	
H3	1.334536	0.060513	0.117218	0.020*	
C4	1.3429 (2)	0.0912 (2)	0.25546 (13)	0.0177 (4)	
H4	1.451212	0.029817	0.272382	0.021*	
C5	1.2548 (2)	0.1629 (3)	0.32327 (12)	0.0163 (4)	
H5	1.301711	0.149691	0.386746	0.020*	
C6	1.0978 (2)	0.2542 (2)	0.29786 (12)	0.0128 (3)	
H6	1.039430	0.306526	0.343897	0.015*	
C7	0.8535 (2)	0.3634 (2)	0.18179 (12)	0.0109 (3)	
C8	1.0403 (3)	0.2111 (3)	0.03369 (13)	0.0230 (5)	
H8A	1.139556	0.190759	−0.003126	0.034*	
H8B	0.991635	0.320424	0.020737	0.034*	
H8C	0.942754	0.131322	0.017568	0.034*	
C9	0.2906 (2)	0.6074 (2)	0.38067 (11)	0.0112 (3)	
C10	0.3975 (2)	0.5094 (2)	0.44575 (12)	0.0136 (3)	
C11	0.3296 (3)	0.4755 (2)	0.52821 (13)	0.0176 (4)	
H11	0.397722	0.406299	0.572177	0.021*	
C12	0.1661 (3)	0.5397 (2)	0.54805 (13)	0.0181 (4)	
H12	0.124588	0.515956	0.605376	0.022*	
C13	0.0628 (2)	0.6389 (2)	0.48400 (13)	0.0165 (4)	
H13	−0.049035	0.684050	0.497503	0.020*	
C14	0.1242 (2)	0.6717 (3)	0.40001 (11)	0.0135 (3)	
H14	0.053015	0.737965	0.355540	0.016*	
C15	0.3493 (2)	0.64320 (19)	0.28939 (12)	0.0113 (3)	
C16	0.5780 (3)	0.4348 (2)	0.42876 (14)	0.0190 (4)	
H16A	0.563507	0.383911	0.367518	0.029*	
H16B	0.615433	0.352757	0.476157	0.029*	
H16C	0.671673	0.519549	0.431646	0.029*	

### Dichloridobis(2-methylbenzamide-κO)zinc(II) (2) Atomic displacement parameters (Å^2^)

**Table:**

	*U*^11^	*U*^22^	*U*^33^	*U*^12^	*U*^13^	*U*^23^
Zn1	0.00830 (8)	0.01066 (9)	0.01290 (9)	0.00019 (8)	0.00197 (6)	0.00037 (9)
Cl1	0.01314 (19)	0.0252 (2)	0.01136 (19)	0.00219 (17)	−0.00077 (14)	0.00050 (17)
Cl2	0.0186 (2)	0.01049 (19)	0.0251 (2)	−0.00277 (17)	0.00381 (18)	0.00124 (18)
O1	0.0107 (6)	0.0098 (6)	0.0171 (6)	0.0007 (5)	−0.0001 (5)	−0.0020 (5)
O2	0.0082 (5)	0.0214 (6)	0.0143 (5)	−0.0029 (6)	0.0020 (4)	−0.0005 (6)
N1	0.0123 (7)	0.0113 (7)	0.0318 (10)	0.0008 (6)	−0.0042 (7)	−0.0047 (7)
N2	0.0094 (6)	0.0219 (9)	0.0117 (7)	−0.0016 (6)	0.0021 (5)	0.0013 (6)
C1	0.0093 (7)	0.0084 (7)	0.0136 (8)	−0.0015 (6)	0.0022 (6)	−0.0001 (6)
C2	0.0132 (7)	0.0134 (8)	0.0138 (7)	−0.0011 (7)	0.0036 (6)	0.0006 (8)
C3	0.0145 (8)	0.0171 (8)	0.0207 (9)	0.0013 (7)	0.0077 (7)	−0.0026 (8)
C4	0.0127 (8)	0.0165 (8)	0.0237 (10)	0.0034 (7)	0.0012 (7)	0.0018 (8)
C5	0.0157 (8)	0.0168 (9)	0.0153 (8)	0.0016 (8)	−0.0015 (6)	0.0019 (8)
C6	0.0128 (8)	0.0123 (7)	0.0137 (8)	−0.0016 (6)	0.0030 (6)	−0.0023 (6)
C7	0.0114 (8)	0.0107 (8)	0.0108 (8)	−0.0005 (6)	0.0018 (6)	0.0016 (6)
C8	0.0220 (9)	0.0320 (13)	0.0154 (9)	0.0030 (8)	0.0043 (7)	0.0001 (8)
C9	0.0099 (7)	0.0122 (7)	0.0117 (8)	−0.0036 (6)	0.0017 (6)	−0.0022 (7)
C10	0.0126 (8)	0.0126 (7)	0.0147 (8)	−0.0019 (6)	−0.0011 (6)	−0.0009 (7)
C11	0.0220 (10)	0.0150 (8)	0.0149 (9)	−0.0045 (7)	−0.0008 (7)	0.0014 (7)
C12	0.0224 (10)	0.0193 (9)	0.0137 (9)	−0.0072 (7)	0.0064 (7)	−0.0015 (7)
C13	0.0140 (8)	0.0200 (9)	0.0164 (8)	−0.0036 (6)	0.0056 (6)	−0.0041 (7)
C14	0.0119 (7)	0.0138 (7)	0.0148 (7)	−0.0013 (8)	0.0015 (5)	−0.0016 (8)
C15	0.0113 (7)	0.0098 (8)	0.0128 (8)	0.0000 (5)	0.0017 (6)	−0.0008 (6)
C16	0.0146 (8)	0.0209 (9)	0.0212 (10)	0.0048 (7)	0.0008 (7)	0.0036 (8)

### Dichloridobis(2-methylbenzamide-κO)zinc(II) (2) Geometric parameters (Å, º)

**Table:**

Zn1—Cl1	2.2340 (4)	C5—H5	0.9500
Zn1—Cl2	2.1947 (5)	C5—C6	1.389 (2)
Zn1—O1	2.0169 (13)	C6—H6	0.9500
Zn1—O2	1.9781 (11)	C8—H8A	0.9800
O1—C7	1.266 (2)	C8—H8B	0.9800
O2—C15	1.2637 (19)	C8—H8C	0.9800
N1—H1A	0.82 (2)	C9—C10	1.405 (2)
N1—H1B	0.858 (19)	C9—C14	1.402 (2)
N1—C7	1.310 (2)	C9—C15	1.486 (2)
N2—H2A	0.847 (18)	C10—C11	1.393 (3)
N2—H2B	0.836 (18)	C10—C16	1.519 (3)
N2—C15	1.313 (2)	C11—H11	0.9500
C1—C2	1.406 (2)	C11—C12	1.384 (3)
C1—C6	1.394 (2)	C12—H12	0.9500
C1—C7	1.490 (2)	C12—C13	1.389 (3)
C2—C3	1.391 (2)	C13—H13	0.9500
C2—C8	1.512 (2)	C13—C14	1.390 (2)
C3—H3	0.9500	C14—H14	0.9500
C3—C4	1.386 (3)	C16—H16A	0.9800
C4—H4	0.9500	C16—H16B	0.9800
C4—C5	1.389 (3)	C16—H16C	0.9800
			
Cl2—Zn1—Cl1	125.120 (19)	N1—C7—C1	118.01 (16)
O1—Zn1—Cl1	107.22 (4)	C2—C8—H8A	109.5
O1—Zn1—Cl2	102.18 (4)	C2—C8—H8B	109.5
O2—Zn1—Cl1	108.84 (3)	C2—C8—H8C	109.5
O2—Zn1—Cl2	107.52 (4)	H8A—C8—H8B	109.5
O2—Zn1—O1	103.91 (5)	H8A—C8—H8C	109.5
C7—O1—Zn1	130.95 (12)	H8B—C8—H8C	109.5
C15—O2—Zn1	131.79 (10)	C10—C9—C15	121.02 (15)
H1A—N1—H1B	119 (2)	C14—C9—C10	120.54 (16)
C7—N1—H1A	118.0 (15)	C14—C9—C15	118.44 (15)
C7—N1—H1B	121.5 (16)	C9—C10—C16	123.15 (16)
H2A—N2—H2B	118 (2)	C11—C10—C9	117.69 (17)
C15—N2—H2A	119.8 (14)	C11—C10—C16	119.12 (16)
C15—N2—H2B	121.5 (15)	C10—C11—H11	119.0
C2—C1—C7	121.28 (15)	C12—C11—C10	122.04 (17)
C6—C1—C2	120.96 (16)	C12—C11—H11	119.0
C6—C1—C7	117.75 (16)	C11—C12—H12	120.0
C1—C2—C8	122.60 (16)	C11—C12—C13	119.91 (18)
C3—C2—C1	117.57 (15)	C13—C12—H12	120.0
C3—C2—C8	119.84 (16)	C12—C13—H13	120.2
C2—C3—H3	119.1	C12—C13—C14	119.52 (18)
C4—C3—C2	121.86 (17)	C14—C13—H13	120.2
C4—C3—H3	119.1	C9—C14—H14	119.9
C3—C4—H4	120.1	C13—C14—C9	120.27 (16)
C3—C4—C5	119.86 (17)	C13—C14—H14	119.9
C5—C4—H4	120.1	O2—C15—N2	122.81 (16)
C4—C5—H5	120.2	O2—C15—C9	119.34 (14)
C4—C5—C6	119.70 (16)	N2—C15—C9	117.86 (15)
C6—C5—H5	120.2	C10—C16—H16A	109.5
C1—C6—H6	120.0	C10—C16—H16B	109.5
C5—C6—C1	120.02 (16)	C10—C16—H16C	109.5
C5—C6—H6	120.0	H16A—C16—H16B	109.5
O1—C7—N1	122.80 (17)	H16A—C16—H16C	109.5
O1—C7—C1	119.18 (15)	H16B—C16—H16C	109.5
			
Zn1—O1—C7—N1	−6.3 (3)	C7—C1—C6—C5	177.87 (16)
Zn1—O1—C7—C1	174.92 (12)	C8—C2—C3—C4	−179.24 (17)
Zn1—O2—C15—N2	−11.9 (3)	C9—C10—C11—C12	−2.2 (3)
Zn1—O2—C15—C9	168.30 (12)	C10—C9—C14—C13	−0.1 (3)
C1—C2—C3—C4	0.9 (3)	C10—C9—C15—O2	−38.3 (2)
C2—C1—C6—C5	−1.9 (3)	C10—C9—C15—N2	141.92 (17)
C2—C1—C7—O1	−119.46 (19)	C10—C11—C12—C13	1.1 (3)
C2—C1—C7—N1	61.7 (2)	C11—C12—C13—C14	0.6 (3)
C2—C3—C4—C5	−0.7 (3)	C12—C13—C14—C9	−1.1 (3)
C3—C4—C5—C6	−0.8 (3)	C14—C9—C10—C11	1.7 (3)
C4—C5—C6—C1	2.1 (3)	C14—C9—C10—C16	179.43 (17)
C6—C1—C2—C3	0.5 (3)	C14—C9—C15—O2	142.68 (18)
C6—C1—C2—C8	−179.44 (18)	C14—C9—C15—N2	−37.1 (2)
C6—C1—C7—O1	60.7 (2)	C15—C9—C10—C11	−177.30 (16)
C6—C1—C7—N1	−118.08 (19)	C15—C9—C10—C16	0.4 (3)
C7—C1—C2—C3	−179.36 (16)	C15—C9—C14—C13	178.94 (16)
C7—C1—C2—C8	0.7 (3)	C16—C10—C11—C12	179.92 (17)

### Dichloridobis(2-methylbenzamide-κO)zinc(II) (2) Hydrogen-bond geometry (Å, º)

**Table:**

*D*—H···*A*	*D*—H	H···*A*	*D*···*A*	*D*—H···*A*
N1—H1*A*···Cl2^i^	0.82 (2)	2.57 (2)	3.2916 (17)	147 (2)
N1—H1*B*···Cl1	0.86 (2)	2.54 (2)	3.3077 (17)	150 (2)
N2—H2*A*···Cl1	0.85 (2)	2.52 (2)	3.2667 (16)	148 (2)
N2—H2*B*···O1^ii^	0.84 (2)	2.14 (2)	2.949 (2)	163 (2)

Symmetry codes: (i) *x*, *y*−1, *z*; (ii) *x*−1, *y*, *z*.

### Dichloridobis(3-methylbenzamide-κO)zinc(II) (3) Crystal data

**Table:**

[ZnCl_2_(C_8_H_9_NO)_2_]	*F*(000) = 832
*M**_r_* = 406.59	*D*_x_ = 1.519 Mg m^−^^3^
Monoclinic, *C*2/*c*	Mo *K*α radiation, λ = 0.71073 Å
*a* = 13.9452 (11) Å	Cell parameters from 6211 reflections
*b* = 18.9742 (16) Å	θ = 3.1–28.7°
*c* = 7.0651 (6) Å	µ = 1.69 mm^−^^1^
β = 108.021 (2)°	*T* = 100 K
*V* = 1777.7 (3) Å^3^	Needle, clear light colourless
*Z* = 4	0.42 × 0.14 × 0.14 mm

### Dichloridobis(3-methylbenzamide-κO)zinc(II) (3) Data collection

**Table:**

Bruker APEXII CCD diffractometer	2295 independent reflections
Radiation source: sealed tube	2023 reflections with *I* > 2σ(*I*)
Graphite monochromator	*R*_int_ = 0.027
Detector resolution: 8 pixels mm^-1^	θ_max_ = 28.7°, θ_min_ = 1.9°
ω and φ scans	*h* = −18→18
Absorption correction: multi-scan (SADABS; Krause *et al.*, 2015)	*k* = −25→25
*T*_min_ = 0.620, *T*_max_ = 0.746	*l* = −9→9
12177 measured reflections	

### Dichloridobis(3-methylbenzamide-κO)zinc(II) (3) Refinement

**Table:**

Refinement on *F*^2^	Primary atom site location: dual
Least-squares matrix: full	Hydrogen site location: mixed
*R*[*F*^2^ > 2σ(*F*^2^)] = 0.022	H atoms treated by a mixture of independent and constrained refinement
*wR*(*F*^2^) = 0.059	*w* = 1/[σ^2^(*F*_o_^2^) + (0.0284*P*)^2^ + 1.5576*P*] where *P* = (*F*_o_^2^ + 2*F*_c_^2^)/3
*S* = 1.05	(Δ/σ)_max_ = 0.001
2295 reflections	Δρ_max_ = 0.39 e Å^−^^3^
113 parameters	Δρ_min_ = −0.26 e Å^−^^3^
17 restraints	

### Dichloridobis(3-methylbenzamide-κO)zinc(II) (3) Special details

**Table:**

Geometry. All esds (except the esd in the dihedral angle between two l.s. planes) are estimated using the full covariance matrix. The cell esds are taken into account individually in the estimation of esds in distances, angles and torsion angles; correlations between esds in cell parameters are only used when they are defined by crystal symmetry. An approximate (isotropic) treatment of cell esds is used for estimating esds involving l.s. planes.

### Dichloridobis(3-methylbenzamide-κO)zinc(II) (3) Fractional atomic coordinates and isotropic or equivalent isotropic displacement parameters (Å^2^)

**Table:**

	*x*	*y*	*z*	*U*_iso_*/*U*_eq_	Occ. (<1)
Zn1	0.500000	0.38624 (2)	0.750000	0.01707 (8)	
Cl1	0.62434 (3)	0.44400 (2)	0.97242 (5)	0.02080 (9)	
O1	0.56261 (8)	0.31701 (5)	0.61793 (15)	0.0204 (2)	
N1	0.61233 (10)	0.38460 (7)	0.40319 (19)	0.0202 (3)	
H1A	0.5980 (15)	0.4225 (9)	0.452 (3)	0.030*	
H1B	0.6324 (15)	0.3898 (10)	0.302 (3)	0.030*	
C1	0.62069 (10)	0.25740 (7)	0.3812 (2)	0.0158 (3)	
C2	0.61038 (10)	0.19345 (8)	0.4691 (2)	0.0180 (3)	
H2	0.588635	0.193263	0.583965	0.022*	
C3	0.63116 (12)	0.12987 (8)	0.3927 (2)	0.0221 (3)	
C4	0.66207 (12)	0.13155 (8)	0.2221 (2)	0.0247 (3)	
H4	0.676788	0.088681	0.167359	0.030*	
C5	0.67144 (12)	0.19491 (8)	0.1323 (2)	0.0234 (3)	
H5	0.691823	0.195033	0.015859	0.028*	
C6	0.65141 (11)	0.25802 (8)	0.2104 (2)	0.0187 (3)	
H6	0.658428	0.301380	0.148814	0.022*	
C7	0.59708 (10)	0.32279 (7)	0.4738 (2)	0.0158 (3)	
C8	0.62193 (14)	0.06111 (9)	0.4921 (3)	0.0332 (4)	
H8A	0.630921	0.069427	0.633494	0.050*	0.54 (2)
H8B	0.673809	0.028337	0.478992	0.050*	0.54 (2)
H8C	0.555047	0.040823	0.428655	0.050*	0.54 (2)
H8D	0.608931	0.022964	0.393933	0.050*	0.46 (2)
H8E	0.566042	0.064055	0.548436	0.050*	0.46 (2)
H8F	0.684804	0.051568	0.598773	0.050*	0.46 (2)

### Dichloridobis(3-methylbenzamide-κO)zinc(II) (3) Atomic displacement parameters (Å^2^)

**Table:**

	*U*^11^	*U*^22^	*U*^33^	*U*^12^	*U*^13^	*U*^23^
Zn1	0.02315 (13)	0.01516 (12)	0.01467 (12)	0.000	0.00842 (9)	0.000
Cl1	0.02732 (19)	0.01659 (17)	0.01841 (16)	−0.00333 (13)	0.00694 (14)	−0.00035 (12)
O1	0.0265 (6)	0.0175 (5)	0.0197 (5)	0.0021 (4)	0.0110 (4)	−0.0008 (4)
N1	0.0259 (7)	0.0152 (6)	0.0214 (6)	0.0015 (5)	0.0099 (5)	−0.0002 (5)
C1	0.0121 (6)	0.0173 (6)	0.0165 (6)	0.0004 (5)	0.0023 (5)	−0.0016 (5)
C2	0.0148 (7)	0.0199 (7)	0.0184 (6)	0.0015 (5)	0.0038 (5)	0.0002 (5)
C3	0.0189 (7)	0.0171 (7)	0.0283 (8)	0.0006 (5)	0.0046 (6)	0.0001 (6)
C4	0.0234 (8)	0.0204 (7)	0.0306 (8)	0.0008 (6)	0.0089 (6)	−0.0079 (6)
C5	0.0216 (7)	0.0277 (8)	0.0233 (7)	−0.0005 (6)	0.0103 (6)	−0.0057 (6)
C6	0.0170 (7)	0.0196 (7)	0.0196 (6)	−0.0007 (5)	0.0058 (5)	−0.0006 (5)
C7	0.0130 (6)	0.0168 (6)	0.0157 (6)	0.0004 (5)	0.0015 (5)	−0.0008 (5)
C8	0.0398 (10)	0.0178 (8)	0.0439 (10)	0.0011 (7)	0.0156 (8)	0.0039 (7)

### Dichloridobis(3-methylbenzamide-κO)zinc(II) (3) Geometric parameters (Å, º)

**Table:**

Zn1—Cl1	2.2341 (4)	C3—C4	1.400 (2)
Zn1—Cl1^i^	2.2341 (4)	C3—C8	1.506 (2)
Zn1—O1^i^	1.9652 (10)	C4—H4	0.9500
Zn1—O1	1.9652 (10)	C4—C5	1.384 (2)
O1—C7	1.2581 (16)	C5—H5	0.9500
N1—H1A	0.847 (15)	C5—C6	1.383 (2)
N1—H1B	0.848 (15)	C6—H6	0.9500
N1—C7	1.3173 (18)	C8—H8A	0.9800
C1—C2	1.3906 (19)	C8—H8B	0.9800
C1—C6	1.3998 (19)	C8—H8C	0.9800
C1—C7	1.4863 (19)	C8—H8D	0.9800
C2—H2	0.9500	C8—H8E	0.9800
C2—C3	1.388 (2)	C8—H8F	0.9800
			
Cl1—Zn1—Cl1^i^	121.25 (2)	C5—C6—C1	119.36 (14)
O1^i^—Zn1—Cl1	110.86 (3)	C5—C6—H6	120.3
O1—Zn1—Cl1^i^	110.86 (3)	O1—C7—N1	122.08 (13)
O1—Zn1—Cl1	107.44 (3)	O1—C7—C1	118.40 (12)
O1^i^—Zn1—Cl1^i^	107.44 (3)	N1—C7—C1	119.53 (12)
O1^i^—Zn1—O1	96.12 (6)	C3—C8—H8A	109.5
C7—O1—Zn1	131.54 (9)	C3—C8—H8B	109.5
H1A—N1—H1B	114.8 (18)	C3—C8—H8C	109.5
C7—N1—H1A	121.2 (13)	C3—C8—H8D	109.5
C7—N1—H1B	123.8 (13)	C3—C8—H8E	109.5
C2—C1—C6	119.59 (13)	C3—C8—H8F	109.5
C2—C1—C7	117.67 (12)	H8A—C8—H8B	109.5
C6—C1—C7	122.74 (13)	H8A—C8—H8C	109.5
C1—C2—H2	119.3	H8A—C8—H8D	141.1
C3—C2—C1	121.45 (13)	H8A—C8—H8E	56.3
C3—C2—H2	119.3	H8A—C8—H8F	56.3
C2—C3—C4	118.15 (14)	H8B—C8—H8C	109.5
C2—C3—C8	120.88 (15)	H8B—C8—H8D	56.3
C4—C3—C8	120.97 (14)	H8B—C8—H8E	141.1
C3—C4—H4	119.6	H8B—C8—H8F	56.3
C5—C4—C3	120.80 (14)	H8C—C8—H8D	56.3
C5—C4—H4	119.6	H8C—C8—H8E	56.3
C4—C5—H5	119.7	H8C—C8—H8F	141.1
C6—C5—C4	120.65 (14)	H8D—C8—H8E	109.5
C6—C5—H5	119.7	H8D—C8—H8F	109.5
C1—C6—H6	120.3	H8E—C8—H8F	109.5
			
Zn1—O1—C7—N1	12.2 (2)	C3—C4—C5—C6	0.7 (2)
Zn1—O1—C7—C1	−167.56 (9)	C4—C5—C6—C1	−0.5 (2)
C1—C2—C3—C4	−0.7 (2)	C6—C1—C2—C3	0.8 (2)
C1—C2—C3—C8	178.60 (15)	C6—C1—C7—O1	174.61 (13)
C2—C1—C6—C5	−0.3 (2)	C6—C1—C7—N1	−5.2 (2)
C2—C1—C7—O1	−4.73 (19)	C7—C1—C2—C3	−179.79 (13)
C2—C1—C7—N1	175.49 (13)	C7—C1—C6—C5	−179.60 (13)
C2—C3—C4—C5	−0.1 (2)	C8—C3—C4—C5	−179.35 (15)

Symmetry code: (i) −*x*+1, *y*, −*z*+3/2.

### Dichloridobis(3-methylbenzamide-κO)zinc(II) (3) Hydrogen-bond geometry (Å, º)

**Table:**

*D*—H···*A*	*D*—H	H···*A*	*D*···*A*	*D*—H···*A*
N1—H1*A*···Cl1^ii^	0.85 (2)	2.56 (2)	3.2854 (13)	145 (2)
N1—H1*B*···Cl1^iii^	0.85 (2)	2.52 (2)	3.2979 (13)	153 (2)

Symmetry codes: (ii) *x*, −*y*+1, *z*−1/2; (iii) *x*, *y*, *z*−1.

### Dichloridobis(4-methylbenzamide-κO)zinc(II) (4) Crystal data

**Table:**

[ZnCl_2_(C_8_H_9_NO)_2_]	*F*(000) = 832
*M**_r_* = 406.59	*D*_x_ = 1.537 Mg m^−^^3^
Monoclinic, *P*2_1_/*c*	Mo *K*α radiation, λ = 0.71073 Å
*a* = 6.8376 (4) Å	Cell parameters from 8511 reflections
*b* = 17.2694 (9) Å	θ = 2.4–30.1°
*c* = 14.9856 (7) Å	µ = 1.71 mm^−^^1^
β = 96.893 (2)°	*T* = 100 K
*V* = 1756.73 (16) Å^3^	Needle, clear light colourless
*Z* = 4	0.56 × 0.18 × 0.09 mm

### Dichloridobis(4-methylbenzamide-κO)zinc(II) (4) Data collection

**Table:**

Bruker APEXII CCD diffractometer	5376 independent reflections
Radiation source: sealed tube	4283 reflections with *I* > 2σ(*I*)
Graphite monochromator	*R*_int_ = 0.051
Detector resolution: 8 pixels mm^-1^	θ_max_ = 30.5°, θ_min_ = 1.8°
ω and φ scans	*h* = −9→9
Absorption correction: multi-scan (SADABS; Krause *et al.*, 2015)	*k* = −24→24
*T*_min_ = 0.629, *T*_max_ = 0.746	*l* = −21→21
33806 measured reflections	

### Dichloridobis(4-methylbenzamide-κO)zinc(II) (4) Refinement

**Table:**

Refinement on *F*^2^	Primary atom site location: dual
Least-squares matrix: full	Hydrogen site location: mixed
*R*[*F*^2^ > 2σ(*F*^2^)] = 0.031	H atoms treated by a mixture of independent and constrained refinement
*wR*(*F*^2^) = 0.069	*w* = 1/[σ^2^(*F*_o_^2^) + (0.028*P*)^2^ + 0.9244*P*] where *P* = (*F*_o_^2^ + 2*F*_c_^2^)/3
*S* = 1.01	(Δ/σ)_max_ = 0.001
5376 reflections	Δρ_max_ = 0.46 e Å^−^^3^
222 parameters	Δρ_min_ = −0.32 e Å^−^^3^
4 restraints	

### Dichloridobis(4-methylbenzamide-κO)zinc(II) (4) Special details

**Table:**

Geometry. All esds (except the esd in the dihedral angle between two l.s. planes) are estimated using the full covariance matrix. The cell esds are taken into account individually in the estimation of esds in distances, angles and torsion angles; correlations between esds in cell parameters are only used when they are defined by crystal symmetry. An approximate (isotropic) treatment of cell esds is used for estimating esds involving l.s. planes.

### Dichloridobis(4-methylbenzamide-κO)zinc(II) (4) Fractional atomic coordinates and isotropic or equivalent isotropic displacement parameters (Å^2^)

**Table:**

	*x*	*y*	*z*	*U*_iso_*/*U*_eq_	
Zn1	0.62040 (3)	0.33576 (2)	0.90851 (2)	0.01257 (6)	
Cl1	0.77285 (6)	0.44248 (3)	0.96142 (3)	0.01698 (9)	
Cl2	0.47211 (6)	0.26784 (3)	1.00645 (3)	0.01897 (9)	
O1	0.80611 (18)	0.27132 (8)	0.85157 (8)	0.0185 (3)	
O2	0.42159 (16)	0.36421 (7)	0.80292 (7)	0.0138 (2)	
N1	0.6780 (2)	0.27321 (10)	0.70593 (9)	0.0173 (3)	
H1A	0.581 (3)	0.3025 (12)	0.7182 (14)	0.026*	
H1B	0.679 (3)	0.2600 (13)	0.6506 (11)	0.026*	
N2	0.1956 (2)	0.41980 (11)	0.87969 (10)	0.0227 (4)	
H2A	0.076 (3)	0.4331 (14)	0.8844 (16)	0.034*	
H2B	0.275 (3)	0.4161 (14)	0.9295 (12)	0.034*	
C1	0.9836 (2)	0.20466 (10)	0.75021 (10)	0.0121 (3)	
C2	1.1478 (2)	0.20012 (10)	0.81520 (10)	0.0135 (3)	
H2	1.145841	0.225037	0.871603	0.016*	
C3	1.3132 (2)	0.15949 (10)	0.79764 (11)	0.0147 (3)	
H3	1.424273	0.157277	0.842184	0.018*	
C4	1.3201 (2)	0.12177 (10)	0.71601 (11)	0.0140 (3)	
C5	1.1545 (2)	0.12644 (10)	0.65131 (11)	0.0149 (3)	
H5	1.156387	0.101115	0.595143	0.018*	
C6	0.9882 (2)	0.16732 (10)	0.66776 (10)	0.0138 (3)	
H6	0.877513	0.169957	0.623050	0.017*	
C7	0.8142 (2)	0.25188 (10)	0.77107 (10)	0.0130 (3)	
C8	1.4980 (3)	0.07558 (11)	0.69831 (12)	0.0200 (4)	
H8A	1.469032	0.020189	0.702178	0.030*	
H8B	1.609350	0.088824	0.743154	0.030*	
H8C	1.531608	0.087642	0.638088	0.030*	
C9	0.1206 (2)	0.40383 (10)	0.71921 (10)	0.0135 (3)	
C10	0.1505 (2)	0.35807 (11)	0.64562 (11)	0.0153 (3)	
H10	0.255602	0.321747	0.650256	0.018*	
C11	0.0270 (3)	0.36541 (11)	0.56531 (11)	0.0189 (4)	
H11	0.047805	0.333358	0.515810	0.023*	
C12	−0.1263 (3)	0.41870 (11)	0.55606 (12)	0.0197 (4)	
C13	−0.1537 (3)	0.46467 (12)	0.62984 (13)	0.0250 (4)	
H13	−0.257293	0.501643	0.624800	0.030*	
C14	−0.0324 (3)	0.45754 (11)	0.71067 (12)	0.0217 (4)	
H14	−0.053924	0.489388	0.760262	0.026*	
C15	0.2540 (2)	0.39488 (10)	0.80440 (10)	0.0131 (3)	
C16	−0.2596 (3)	0.42661 (13)	0.46859 (13)	0.0276 (4)	
H16A	−0.220031	0.389179	0.424975	0.041*	
H16B	−0.249033	0.479199	0.445066	0.041*	
H16C	−0.396095	0.416577	0.478950	0.041*	

### Dichloridobis(4-methylbenzamide-κO)zinc(II) (4) Atomic displacement parameters (Å^2^)

**Table:**

	*U*^11^	*U*^22^	*U*^33^	*U*^12^	*U*^13^	*U*^23^
Zn1	0.01264 (9)	0.01536 (11)	0.00929 (8)	0.00095 (8)	−0.00036 (6)	−0.00125 (7)
Cl1	0.01717 (18)	0.0165 (2)	0.01668 (18)	−0.00171 (16)	−0.00044 (14)	−0.00253 (15)
Cl2	0.01998 (19)	0.0250 (2)	0.01107 (16)	−0.00528 (17)	−0.00144 (14)	0.00284 (16)
O1	0.0177 (6)	0.0255 (7)	0.0120 (5)	0.0070 (5)	0.0000 (4)	−0.0039 (5)
O2	0.0119 (5)	0.0180 (6)	0.0111 (5)	0.0022 (5)	−0.0002 (4)	−0.0012 (4)
N1	0.0164 (7)	0.0238 (9)	0.0112 (6)	0.0065 (6)	−0.0008 (5)	−0.0040 (6)
N2	0.0158 (7)	0.0376 (10)	0.0141 (7)	0.0066 (7)	−0.0002 (6)	−0.0059 (7)
C1	0.0123 (7)	0.0114 (8)	0.0123 (7)	−0.0012 (6)	0.0009 (6)	0.0002 (6)
C2	0.0147 (7)	0.0146 (9)	0.0107 (7)	−0.0008 (6)	−0.0006 (6)	−0.0007 (6)
C3	0.0133 (7)	0.0152 (9)	0.0146 (7)	−0.0004 (6)	−0.0024 (6)	0.0012 (6)
C4	0.0129 (7)	0.0117 (8)	0.0177 (7)	−0.0005 (6)	0.0029 (6)	0.0010 (6)
C5	0.0173 (8)	0.0140 (9)	0.0133 (7)	−0.0001 (7)	0.0018 (6)	−0.0023 (6)
C6	0.0141 (7)	0.0144 (8)	0.0125 (6)	−0.0001 (6)	−0.0003 (6)	−0.0011 (6)
C7	0.0128 (7)	0.0134 (8)	0.0126 (7)	−0.0022 (6)	0.0006 (6)	−0.0007 (6)
C8	0.0151 (8)	0.0196 (10)	0.0250 (8)	0.0023 (7)	0.0012 (7)	−0.0032 (7)
C9	0.0127 (7)	0.0132 (8)	0.0139 (7)	−0.0006 (6)	−0.0015 (6)	−0.0014 (6)
C10	0.0147 (7)	0.0167 (9)	0.0140 (7)	0.0023 (6)	0.0001 (6)	−0.0011 (6)
C11	0.0214 (8)	0.0213 (10)	0.0134 (7)	−0.0010 (7)	−0.0010 (6)	−0.0039 (7)
C12	0.0194 (8)	0.0182 (10)	0.0193 (8)	−0.0021 (7)	−0.0065 (7)	0.0020 (7)
C13	0.0219 (9)	0.0201 (10)	0.0301 (10)	0.0084 (8)	−0.0088 (8)	−0.0032 (8)
C14	0.0221 (9)	0.0195 (10)	0.0218 (8)	0.0060 (7)	−0.0040 (7)	−0.0064 (7)
C15	0.0132 (7)	0.0130 (8)	0.0128 (7)	−0.0016 (6)	0.0001 (6)	−0.0014 (6)
C16	0.0282 (10)	0.0273 (11)	0.0237 (9)	−0.0014 (8)	−0.0121 (8)	0.0023 (8)

### Dichloridobis(4-methylbenzamide-κO)zinc(II) (4) Geometric parameters (Å, º)

**Table:**

Zn1—Cl1	2.2166 (5)	C5—H5	0.9500
Zn1—Cl2	2.2170 (5)	C5—C6	1.385 (2)
Zn1—O1	1.9592 (12)	C6—H6	0.9500
Zn1—O2	2.0191 (11)	C8—H8A	0.9800
O1—C7	1.2599 (19)	C8—H8B	0.9800
O2—C15	1.265 (2)	C8—H8C	0.9800
N1—H1A	0.869 (16)	C9—C10	1.392 (2)
N1—H1B	0.861 (15)	C9—C14	1.393 (2)
N1—C7	1.318 (2)	C9—C15	1.485 (2)
N2—H2A	0.858 (16)	C10—H10	0.9500
N2—H2B	0.871 (16)	C10—C11	1.391 (2)
N2—C15	1.313 (2)	C11—H11	0.9500
C1—C2	1.398 (2)	C11—C12	1.389 (3)
C1—C6	1.397 (2)	C12—C13	1.392 (3)
C1—C7	1.480 (2)	C12—C16	1.510 (2)
C2—H2	0.9500	C13—H13	0.9500
C2—C3	1.382 (2)	C13—C14	1.389 (2)
C3—H3	0.9500	C14—H14	0.9500
C3—C4	1.392 (2)	C16—H16A	0.9800
C4—C5	1.402 (2)	C16—H16B	0.9800
C4—C8	1.505 (2)	C16—H16C	0.9800
			
Cl1—Zn1—Cl2	115.836 (17)	N1—C7—C1	119.92 (14)
O1—Zn1—Cl1	109.06 (4)	C4—C8—H8A	109.5
O1—Zn1—Cl2	111.08 (4)	C4—C8—H8B	109.5
O1—Zn1—O2	101.98 (5)	C4—C8—H8C	109.5
O2—Zn1—Cl1	108.75 (4)	H8A—C8—H8B	109.5
O2—Zn1—Cl2	109.22 (4)	H8A—C8—H8C	109.5
C7—O1—Zn1	132.35 (11)	H8B—C8—H8C	109.5
C15—O2—Zn1	127.89 (10)	C10—C9—C14	119.06 (15)
H1A—N1—H1B	117 (2)	C10—C9—C15	119.24 (15)
C7—N1—H1A	119.6 (15)	C14—C9—C15	121.69 (15)
C7—N1—H1B	123.4 (15)	C9—C10—H10	119.9
H2A—N2—H2B	117 (2)	C11—C10—C9	120.14 (16)
C15—N2—H2A	123.2 (16)	C11—C10—H10	119.9
C15—N2—H2B	119.3 (16)	C10—C11—H11	119.4
C2—C1—C7	117.86 (14)	C12—C11—C10	121.30 (16)
C6—C1—C2	119.24 (15)	C12—C11—H11	119.4
C6—C1—C7	122.85 (14)	C11—C12—C13	118.05 (15)
C1—C2—H2	119.9	C11—C12—C16	121.06 (17)
C3—C2—C1	120.19 (15)	C13—C12—C16	120.89 (17)
C3—C2—H2	119.9	C12—C13—H13	119.4
C2—C3—H3	119.3	C14—C13—C12	121.28 (17)
C2—C3—C4	121.31 (14)	C14—C13—H13	119.4
C4—C3—H3	119.3	C9—C14—H14	119.9
C3—C4—C5	118.12 (15)	C13—C14—C9	120.16 (17)
C3—C4—C8	121.10 (14)	C13—C14—H14	119.9
C5—C4—C8	120.77 (15)	O2—C15—N2	121.42 (14)
C4—C5—H5	119.4	O2—C15—C9	119.51 (14)
C6—C5—C4	121.21 (15)	N2—C15—C9	119.07 (15)
C6—C5—H5	119.4	C12—C16—H16A	109.5
C1—C6—H6	120.0	C12—C16—H16B	109.5
C5—C6—C1	119.92 (14)	C12—C16—H16C	109.5
C5—C6—H6	120.0	H16A—C16—H16B	109.5
O1—C7—N1	121.75 (16)	H16A—C16—H16C	109.5
O1—C7—C1	118.33 (14)	H16B—C16—H16C	109.5
			
Zn1—O1—C7—N1	−2.3 (3)	C7—C1—C6—C5	177.51 (16)
Zn1—O1—C7—C1	176.97 (12)	C8—C4—C5—C6	178.48 (17)
Zn1—O2—C15—N2	7.0 (3)	C9—C10—C11—C12	0.8 (3)
Zn1—O2—C15—C9	−173.68 (11)	C10—C9—C14—C13	0.4 (3)
C1—C2—C3—C4	−0.6 (3)	C10—C9—C15—O2	20.4 (2)
C2—C1—C6—C5	0.0 (3)	C10—C9—C15—N2	−160.32 (18)
C2—C1—C7—O1	−15.5 (2)	C10—C11—C12—C13	−0.2 (3)
C2—C1—C7—N1	163.79 (17)	C10—C11—C12—C16	179.77 (18)
C2—C3—C4—C5	0.4 (3)	C11—C12—C13—C14	−0.3 (3)
C2—C3—C4—C8	−178.10 (16)	C12—C13—C14—C9	0.2 (3)
C3—C4—C5—C6	0.0 (3)	C14—C9—C10—C11	−0.9 (3)
C4—C5—C6—C1	−0.2 (3)	C14—C9—C15—O2	−158.86 (17)
C6—C1—C2—C3	0.4 (3)	C14—C9—C15—N2	20.5 (3)
C6—C1—C7—O1	166.97 (16)	C15—C9—C10—C11	179.83 (16)
C6—C1—C7—N1	−13.8 (3)	C15—C9—C14—C13	179.64 (18)
C7—C1—C2—C3	−177.27 (16)	C16—C12—C13—C14	179.70 (19)

### Dichloridobis(4-methylbenzamide-κO)zinc(II) (4) Hydrogen-bond geometry (Å, º)

**Table:**

*D*—H···*A*	*D*—H	H···*A*	*D*···*A*	*D*—H···*A*
N1—H1*A*···O2	0.87 (2)	2.07 (2)	2.8753 (19)	154 (2)
N1—H1*B*···Cl2^i^	0.86 (2)	2.49 (2)	3.2265 (14)	145 (2)
N2—H2*A*···Cl1^ii^	0.86 (2)	2.50 (2)	3.2956 (16)	155 (2)
N2—H2*B*···Cl2	0.87 (2)	3.05 (2)	3.6341 (17)	126 (2)

Symmetry codes: (i) *x*, −*y*+1/2, *z*−1/2; (ii) *x*−1, *y*, *z*.

### Dichloridobis(4-hydroxybenzamide-κO)zinc(II) (5) Crystal data

**Table:**

[ZnCl_2_(C_7_H_7_NO_2_)_2_]	*F*(000) = 832
*M**_r_* = 410.54	*D*_x_ = 1.696 Mg m^−^^3^
Monoclinic, *C**c*	Mo *K*α radiation, λ = 0.71073 Å
*a* = 7.0532 (6) Å	Cell parameters from 5843 reflections
*b* = 21.3776 (17) Å	θ = 2.7–27.6°
*c* = 11.1181 (9) Å	µ = 1.88 mm^−^^1^
β = 106.477 (2)°	*T* = 100 K
*V* = 1607.5 (2) Å^3^	Block, clear light colourless
*Z* = 4	0.15 × 0.09 × 0.07 mm

### Dichloridobis(4-hydroxybenzamide-κO)zinc(II) (5) Data collection

**Table:**

Bruker APEXII CCD diffractometer	4168 independent reflections
Radiation source: sealed tube	3809 reflections with *I* > 2σ(*I*)
Graphite monochromator	*R*_int_ = 0.042
Detector resolution: 8 pixels mm^-1^	θ_max_ = 28.7°, θ_min_ = 1.9°
ω and φ scans	*h* = −9→9
Absorption correction: multi-scan (SADABS; Krause *et al.*, 2015)	*k* = −28→28
*T*_min_ = 0.673, *T*_max_ = 0.746	*l* = −15→15
17255 measured reflections	

### Dichloridobis(4-hydroxybenzamide-κO)zinc(II) (5) Refinement

**Table:**

Refinement on *F*^2^	Hydrogen site location: mixed
Least-squares matrix: full	H atoms treated by a mixture of independent and constrained refinement
*R*[*F*^2^ > 2σ(*F*^2^)] = 0.030	*w* = 1/[σ^2^(*F*_o_^2^) + (0.023*P*)^2^ + 0.9687*P*] where *P* = (*F*_o_^2^ + 2*F*_c_^2^)/3
*wR*(*F*^2^) = 0.065	(Δ/σ)_max_ < 0.001
*S* = 1.05	Δρ_max_ = 0.46 e Å^−^^3^
4168 reflections	Δρ_min_ = −0.29 e Å^−^^3^
227 parameters	Absolute structure: Refined as an inversion twin
8 restraints	Absolute structure parameter: 0.024 (13)
Primary atom site location: dual	

### Dichloridobis(4-hydroxybenzamide-κO)zinc(II) (5) Special details

**Table:**

Geometry. All esds (except the esd in the dihedral angle between two l.s. planes) are estimated using the full covariance matrix. The cell esds are taken into account individually in the estimation of esds in distances, angles and torsion angles; correlations between esds in cell parameters are only used when they are defined by crystal symmetry. An approximate (isotropic) treatment of cell esds is used for estimating esds involving l.s. planes.
Refinement. Refined as a 2-component inversion twin.

### Dichloridobis(4-hydroxybenzamide-κO)zinc(II) (5) Fractional atomic coordinates and isotropic or equivalent isotropic displacement parameters (Å^2^)

**Table:**

	*x*	*y*	*z*	*U*_iso_*/*U*_eq_	
Zn1	0.53881 (5)	0.85209 (2)	0.23775 (4)	0.01454 (12)	
Cl1	0.73319 (15)	0.92222 (5)	0.18199 (9)	0.0198 (2)	
Cl2	0.21825 (15)	0.86831 (5)	0.14477 (10)	0.0210 (2)	
O1	0.5952 (4)	0.84920 (13)	0.4225 (3)	0.0171 (6)	
O2	0.6040 (5)	0.76660 (13)	0.2016 (3)	0.0202 (6)	
O3	0.7811 (5)	0.80301 (14)	1.0011 (3)	0.0203 (7)	
H3	0.833 (7)	0.828 (2)	1.059 (4)	0.030*	
O4	0.6608 (5)	0.49561 (14)	−0.0004 (3)	0.0260 (7)	
H4	0.681 (8)	0.4641 (19)	0.041 (5)	0.039*	
N1	0.6218 (6)	0.95087 (17)	0.4782 (3)	0.0188 (8)	
H1A	0.606 (7)	0.960 (2)	0.401 (3)	0.028*	
H1B	0.644 (7)	0.9847 (17)	0.524 (4)	0.028*	
N2	0.7170 (6)	0.72093 (18)	0.3908 (3)	0.0203 (8)	
H2A	0.711 (8)	0.7563 (16)	0.422 (5)	0.030*	
H2B	0.757 (8)	0.6873 (17)	0.428 (4)	0.030*	
C1	0.6588 (6)	0.87096 (18)	0.6380 (4)	0.0129 (8)	
C2	0.7355 (6)	0.91117 (19)	0.7389 (4)	0.0154 (8)	
H2	0.759638	0.953737	0.723612	0.018*	
C3	0.7767 (6)	0.88968 (19)	0.8609 (4)	0.0153 (8)	
H3A	0.829009	0.917405	0.929006	0.018*	
C4	0.7414 (6)	0.8275 (2)	0.8836 (4)	0.0153 (8)	
C5	0.6645 (6)	0.78625 (19)	0.7834 (4)	0.0157 (8)	
H5	0.641179	0.743710	0.799426	0.019*	
C6	0.6234 (6)	0.80752 (19)	0.6626 (4)	0.0156 (8)	
H6	0.570865	0.779649	0.594761	0.019*	
C7	0.6229 (6)	0.89057 (18)	0.5072 (4)	0.0135 (8)	
C8	0.6692 (6)	0.65874 (18)	0.2021 (4)	0.0129 (8)	
C9	0.7129 (6)	0.60210 (19)	0.2654 (4)	0.0153 (8)	
H9	0.745040	0.601421	0.354333	0.018*	
C10	0.7104 (6)	0.54637 (19)	0.2002 (4)	0.0169 (8)	
H10	0.738364	0.507780	0.244024	0.020*	
C11	0.6661 (6)	0.5479 (2)	0.0692 (4)	0.0165 (8)	
C12	0.6224 (6)	0.6047 (2)	0.0046 (4)	0.0178 (9)	
H12	0.592215	0.605752	−0.084269	0.021*	
C13	0.6238 (6)	0.65941 (19)	0.0717 (4)	0.0154 (8)	
H13	0.593257	0.697989	0.028103	0.018*	
C14	0.6644 (6)	0.71888 (18)	0.2678 (4)	0.0137 (8)	

### Dichloridobis(4-hydroxybenzamide-κO)zinc(II) (5) Atomic displacement parameters (Å^2^)

**Table:**

	*U*^11^	*U*^22^	*U*^33^	*U*^12^	*U*^13^	*U*^23^
Zn1	0.0211 (2)	0.0112 (2)	0.01033 (19)	0.0013 (2)	0.00287 (15)	−0.0002 (2)
Cl1	0.0282 (5)	0.0143 (5)	0.0176 (5)	−0.0022 (4)	0.0078 (4)	0.0003 (4)
Cl2	0.0213 (5)	0.0154 (5)	0.0225 (5)	0.0004 (4)	0.0003 (4)	0.0000 (4)
O1	0.0246 (15)	0.0157 (15)	0.0100 (14)	0.0000 (12)	0.0035 (11)	−0.0003 (11)
O2	0.0332 (17)	0.0102 (15)	0.0194 (15)	0.0024 (12)	0.0109 (13)	0.0015 (11)
O3	0.0308 (17)	0.0190 (16)	0.0090 (14)	−0.0028 (13)	0.0022 (12)	0.0018 (11)
O4	0.0412 (19)	0.0116 (16)	0.0236 (17)	0.0012 (14)	0.0068 (15)	−0.0021 (12)
N1	0.031 (2)	0.0137 (19)	0.0106 (17)	0.0004 (16)	0.0046 (15)	0.0008 (13)
N2	0.032 (2)	0.0143 (19)	0.0137 (18)	0.0036 (17)	0.0043 (16)	−0.0014 (14)
C1	0.014 (2)	0.0111 (18)	0.0125 (18)	0.0009 (15)	0.0028 (15)	−0.0019 (15)
C2	0.018 (2)	0.012 (2)	0.017 (2)	0.0015 (15)	0.0071 (16)	−0.0013 (15)
C3	0.020 (2)	0.014 (2)	0.0111 (19)	0.0004 (16)	0.0025 (15)	−0.0033 (14)
C4	0.014 (2)	0.022 (2)	0.0099 (18)	0.0026 (16)	0.0039 (15)	0.0029 (15)
C5	0.018 (2)	0.013 (2)	0.016 (2)	−0.0004 (16)	0.0062 (15)	0.0012 (15)
C6	0.0155 (19)	0.015 (2)	0.016 (2)	−0.0007 (16)	0.0039 (16)	−0.0026 (16)
C7	0.0138 (19)	0.014 (2)	0.0132 (19)	−0.0013 (15)	0.0052 (15)	−0.0012 (15)
C8	0.0136 (18)	0.0098 (19)	0.0148 (19)	−0.0006 (14)	0.0035 (15)	0.0002 (14)
C9	0.018 (2)	0.012 (2)	0.0148 (19)	−0.0005 (16)	0.0037 (16)	0.0013 (15)
C10	0.021 (2)	0.0115 (19)	0.018 (2)	0.0009 (16)	0.0044 (16)	0.0023 (15)
C11	0.019 (2)	0.014 (2)	0.017 (2)	−0.0016 (16)	0.0038 (16)	−0.0024 (15)
C12	0.020 (2)	0.021 (2)	0.0118 (19)	−0.0001 (17)	0.0022 (16)	−0.0021 (16)
C13	0.0163 (19)	0.013 (2)	0.016 (2)	−0.0018 (15)	0.0033 (15)	0.0020 (15)
C14	0.0148 (18)	0.0110 (19)	0.016 (2)	−0.0023 (15)	0.0061 (15)	0.0014 (15)

### Dichloridobis(4-hydroxybenzamide-κO)zinc(II) (5) Geometric parameters (Å, º)

**Table:**

Zn1—Cl1	2.2347 (11)	C2—H2	0.9500
Zn1—Cl2	2.2305 (11)	C2—C3	1.383 (6)
Zn1—O1	1.980 (3)	C3—H3A	0.9500
Zn1—O2	1.954 (3)	C3—C4	1.388 (6)
O1—C7	1.266 (5)	C4—C5	1.404 (5)
O2—C14	1.259 (5)	C5—H5	0.9500
O3—H3	0.84 (3)	C5—C6	1.369 (6)
O3—C4	1.361 (5)	C6—H6	0.9500
O4—H4	0.80 (3)	C8—C9	1.390 (5)
O4—C11	1.353 (5)	C8—C13	1.394 (6)
N1—H1A	0.86 (3)	C8—C14	1.483 (5)
N1—H1B	0.87 (3)	C9—H9	0.9500
N1—C7	1.328 (5)	C9—C10	1.392 (6)
N2—H2A	0.84 (3)	C10—H10	0.9500
N2—H2B	0.84 (3)	C10—C11	1.400 (6)
N2—C14	1.313 (5)	C11—C12	1.401 (6)
C1—C2	1.395 (5)	C12—H12	0.9500
C1—C6	1.419 (5)	C12—C13	1.386 (6)
C1—C7	1.465 (5)	C13—H13	0.9500
			
Cl2—Zn1—Cl1	112.84 (4)	C6—C5—C4	119.8 (4)
O1—Zn1—Cl1	110.51 (9)	C6—C5—H5	120.1
O1—Zn1—Cl2	111.39 (9)	C1—C6—H6	119.8
O2—Zn1—Cl1	111.83 (10)	C5—C6—C1	120.4 (4)
O2—Zn1—Cl2	108.48 (10)	C5—C6—H6	119.8
O2—Zn1—O1	101.21 (12)	O1—C7—N1	120.6 (4)
C7—O1—Zn1	133.9 (3)	O1—C7—C1	119.0 (4)
C14—O2—Zn1	134.3 (3)	N1—C7—C1	120.4 (4)
C4—O3—H3	115 (4)	C9—C8—C13	119.2 (4)
C11—O4—H4	113 (4)	C9—C8—C14	122.6 (4)
H1A—N1—H1B	111 (5)	C13—C8—C14	118.2 (4)
C7—N1—H1A	117 (3)	C8—C9—H9	119.5
C7—N1—H1B	132 (3)	C8—C9—C10	120.9 (4)
H2A—N2—H2B	128 (5)	C10—C9—H9	119.5
C14—N2—H2A	116 (4)	C9—C10—H10	120.4
C14—N2—H2B	116 (4)	C9—C10—C11	119.2 (4)
C2—C1—C6	118.9 (4)	C11—C10—H10	120.4
C2—C1—C7	122.7 (4)	O4—C11—C10	122.5 (4)
C6—C1—C7	118.3 (3)	O4—C11—C12	117.2 (4)
C1—C2—H2	119.7	C10—C11—C12	120.3 (4)
C3—C2—C1	120.6 (4)	C11—C12—H12	120.4
C3—C2—H2	119.7	C13—C12—C11	119.3 (4)
C2—C3—H3A	120.1	C13—C12—H12	120.4
C2—C3—C4	119.9 (4)	C8—C13—H13	119.5
C4—C3—H3A	120.1	C12—C13—C8	121.1 (4)
O3—C4—C3	123.0 (4)	C12—C13—H13	119.5
O3—C4—C5	116.6 (4)	O2—C14—N2	122.0 (4)
C3—C4—C5	120.4 (4)	O2—C14—C8	117.8 (3)
C4—C5—H5	120.1	N2—C14—C8	120.2 (4)
			
Zn1—O1—C7—N1	−1.8 (6)	C6—C1—C7—N1	168.7 (4)
Zn1—O1—C7—C1	179.0 (3)	C7—C1—C2—C3	−176.6 (4)
Zn1—O2—C14—N2	−7.0 (6)	C7—C1—C6—C5	176.6 (4)
Zn1—O2—C14—C8	171.2 (3)	C8—C9—C10—C11	1.0 (6)
O3—C4—C5—C6	−179.2 (4)	C9—C8—C13—C12	−0.2 (6)
O4—C11—C12—C13	179.3 (4)	C9—C8—C14—O2	−173.0 (4)
C1—C2—C3—C4	−0.1 (6)	C9—C8—C14—N2	5.2 (6)
C2—C1—C6—C5	−0.2 (6)	C9—C10—C11—O4	−180.0 (4)
C2—C1—C7—O1	164.6 (4)	C9—C10—C11—C12	−0.8 (6)
C2—C1—C7—N1	−14.6 (6)	C10—C11—C12—C13	0.1 (6)
C2—C3—C4—O3	179.0 (4)	C11—C12—C13—C8	0.5 (6)
C2—C3—C4—C5	0.2 (6)	C13—C8—C9—C10	−0.5 (6)
C3—C4—C5—C6	−0.3 (6)	C13—C8—C14—O2	5.6 (6)
C4—C5—C6—C1	0.3 (6)	C13—C8—C14—N2	−176.1 (4)
C6—C1—C2—C3	0.1 (6)	C14—C8—C9—C10	178.1 (4)
C6—C1—C7—O1	−12.0 (6)	C14—C8—C13—C12	−179.0 (4)

### Dichloridobis(4-hydroxybenzamide-κO)zinc(II) (5) Hydrogen-bond geometry (Å, º)

**Table:**

*D*—H···*A*	*D*—H	H···*A*	*D*···*A*	*D*—H···*A*
O3—H3···Cl1^i^	0.84 (3)	2.64 (4)	3.322 (3)	140 (5)
O3—H3···Cl2^ii^	0.84 (3)	2.75 (4)	3.349 (3)	130 (4)
O4—H4···Cl2^iii^	0.80 (3)	2.33 (3)	3.131 (3)	175 (6)
N1—H1*A*···Cl1	0.86 (3)	2.93 (4)	3.648 (4)	142 (4)
N1—H1*B*···Cl1^iv^	0.87 (3)	2.61 (3)	3.479 (4)	173 (4)
N2—H2*A*···O1	0.84 (3)	2.15 (3)	2.924 (5)	154 (5)
N2—H2*B*···Cl2^v^	0.84 (3)	2.77 (4)	3.405 (4)	135 (5)

Symmetry codes: (i) *x*, *y*, *z*+1; (ii) *x*+1, *y*, *z*+1; (iii) *x*+1/2, *y*−1/2, *z*; (iv) *x*, −*y*+2, *z*+1/2; (v) *x*+1/2, −*y*+3/2, *z*+1/2.
